# Integrated stress response regulates GDF15 secretion from adipocytes, preferentially suppresses appetite for a high-fat diet and improves obesity

**DOI:** 10.1016/j.isci.2021.103448

**Published:** 2021-11-15

**Authors:** Masato Miyake, Jun Zhang, Akihiro Yasue, Satoshi Hisanaga, Kazue Tsugawa, Hiroshi Sakaue, Miho Oyadomari, Hiroshi Kiyonari, Seiichi Oyadomari

**Affiliations:** 1 Division of Molecular Biology, Institute for Genome Research, Institute of Advanced Medical Sciences, Tokushima University, Tokushima 770-8503, Japan; 2 Diabetes Therapeutics and Research Center, Institute of Advanced Medical Sciences, Tokushima University, Tokushima 770-8503, Japan; 3 Fujii Memorial Institute of Medical Sciences, Institute of Advanced Medical Sciences, Tokushima University, Tokushima 770-8503, Japan; 4 ER Stress Research Institute Inc., Tokushima 770-8503, Japan; 5 Department of Orthodontics and Dentofacial Orthopedics, Institute of Biomedical Sciences, Tokushima University Graduate School, 770-8504, Japan; 6 Department of Orthopaedic Surgery, Faculty of Life Sciences, Kumamoto University, Kumamoto 860-8556, Japan; 7 Department of Nutrition and Metabolism, Institute of Biomedical Sciences, Tokushima University Graduate School, Tokushima 770-8503, Japan; 8 Laboratory for Animal Resources and Genetic Engineering, RIKEN Center for Biosystems Dynamics Research, Kobe 650-0047, Japan

**Keywords:** Biological sciences, Human metabolism, Cell biology

## Abstract

The eIF2α phosphorylation-dependent integrated stress response (ISR) is a signaling pathway that maintains homeostasis in mammalian cells exposed to various stresses. Here, ISR activation in adipocytes improves obesity and diabetes by regulating appetite in a non-cell-autonomous manner. Adipocyte-specific ISR activation using transgenic mice decreases body weight and improves glucose tolerance and obesity induced by a high-fat diet (HFD) via preferential inhibition of HFD intake. The transcriptome analysis of ISR-activated adipose tissue reveals that growth differentiation factor 15 (GDF15) expression is induced by the ISR through the direct regulation of the transcription factors ATF4 and DDIT3. Deficiency in the GDF15 receptor GFRAL abolishes the adipocyte ISR-dependent preferential inhibition of HFD intake and the anti-obesity effects. Pharmacologically, 10(E), 12(Z)-octadecadienoic acid induces ISR-dependent GDF15 expression in adipocytes and decreases the intake of the HFD. Based on our findings the specific activation of the ISR in adipocytes controls the non-cell-autonomous regulation of appetite.

## Highlights

• Activation of ISR in adipocytes suppresses intake of high-fat diet and prevents obesity
• ATF4 and DDIT3 induced by ISR directly regulate GDF15 expression
• GDF15-GFRAL axis mediates the control of appetite for high-fat diet by ISR activation
• One of conjugated linoleic acids induces ISR and GDF15 expression in adipocytes

## Graphical Abstract

**Figure:**
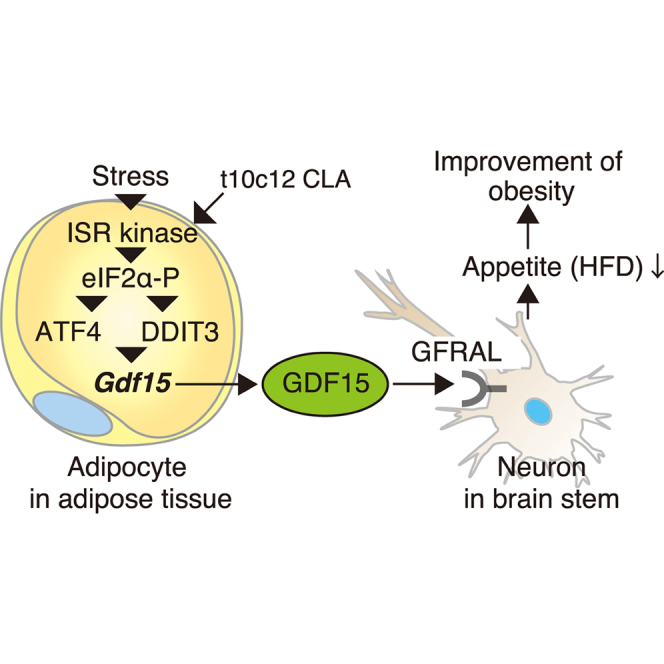

## Introduction

Approximately one-third of the global population is obese or overweight, and obesity is a major risk factor for type 2 diabetes (Roden and Shulman, 2019). Adipose tissue is involved in the development of insulin resistance by regulating energy homeostasis, insulin sensitivity, and lipid and carbohydrate metabolism (Scherer, 2019). In addition to its cell autonomous functions, adipose tissue communicates with other tissues by producing a variety of biologically active molecules that are collectively known as adipokines, including leptin and adiponectin, to maintain systemic metabolic homeostasis (Stern et al., 2016). Therefore, the cellular signaling pathways controlling adipokine production and regulating glucose and lipid metabolism are promising targets for managing obesity and preventing type 2 diabetes.

The integrated stress response (ISR) is a stress response signaling pathway that is initiated upon the phosphorylation of serine-51 in eukaryotic translation initiation factor 2 α (eIF2α). A variety of stresses, including heme depletion, viral infection, endoplasmic reticulum (ER) stress, amino acid depletion, glucose depletion, hypoxia, and hyperosmosis, converge to phosphorylate eIF2α, which activates the ISR to restore cellular homeostasis (Pakos-Zebrucka et al., 2016). As shown in our recent study, four eIF2α kinases, namely, heme-regulated inhibitor (HRI), protein kinase R (PKR), PKR-like ER kinase, (PERK) and general control non-depressible 2 (GCN2), have overlapping functions and cooperatively activate the ISR in response to diverse stress stimuli (Taniuchi et al., 2016). The core events in the ISR are the transient attenuation of the translation of most mRNAs and transcriptional induction mediated by the selective translation of transcription factors, such as activating transcription factor 4 (ATF4). Since protein synthesis is one of the processes that consumes the greatest amount of ATP, a decrease in the rate of translation is likely crucial for cellular adaptations that promote cell survival as a first line of defense. Meanwhile, the expression of genes involved in the cellular stress adaptation is induced as a second line of defense. As the ISR is a homeostatic program, the dysregulation of the ISR may be deleterious for cells and organisms.

ISR-defective mice harboring the homozygous eIF2α^S51A^ mutation die shortly after birth owing to hypoglycemia associated with defective gluconeogenesis (Scheuner et al., 2001). Furthermore, mice carrying a heterozygous eIF2α^S51A^ mutation display impaired glucose metabolism and develop obesity, indicating that the ISR plays an active role in metabolic homeostasis (Scheuner et al., 2005). We and other researchers showed that liver-specific activation of the ISR in the mouse regulates lipid and carbohydrate metabolism in a hepatocyte-autonomous manner (Oyadomari et al., 2008) or systemic glucose homeostasis in a fibroblast growth factor 21 (FGF21)-mediated non-cell autonomous manner (Miyake et al., 2016). Activation of the ISR in the skeletal muscle promotes energy consumption in the brown adipose tissue (BAT) via skeletal muscle-secreted FGF21 (Kim et al., 2013; Miyake et al., 2016). Based on this finding, the ISR regulates cell-autonomous functions and non-cell-autonomous metabolic effects.

In the context of adipocytes, PKR activation was observed in the adipose tissue of obese mice (Nakamura et al., 2010), and activation of PERK or GCN2 in adipocytes is implicated in adipocyte differentiation or stem cell regulation, respectively (Armstrong et al., 2014; Bobrovnikova-Marjon et al., 2012). Thus, the ISR can be activated by metabolic stress in adipose tissue and potentially regulates the fine-tuning of adipocyte functions, but the effects of adipose-specific activation of the ISR on metabolism are not completely understood. In the present study, we explored the role of the ISR in the adipose tissue in regulating metabolism using a transgenic (Tg) mouse model that promotes the adipose tissue-specific activation of the ISR. Activation of the ISR in adipose tissue improved diet-induced obesity and diabetes by inducing the secretion of the adipokine growth differentiation factor 15 (GDF15) to decrease the specific intake of a high-fat diet (HFD). Based on our findings, eIF2α phosphorylation in adipose tissue controls non-cell-autonomous metabolic regulation, providing potential therapeutic targets for obesity and type 2 diabetes.

## Results

### Activation of the ISR in the adipose tissue leads to an HFD-specific body weight loss after adipose-specific activation of the ISR

**Figure 1:**
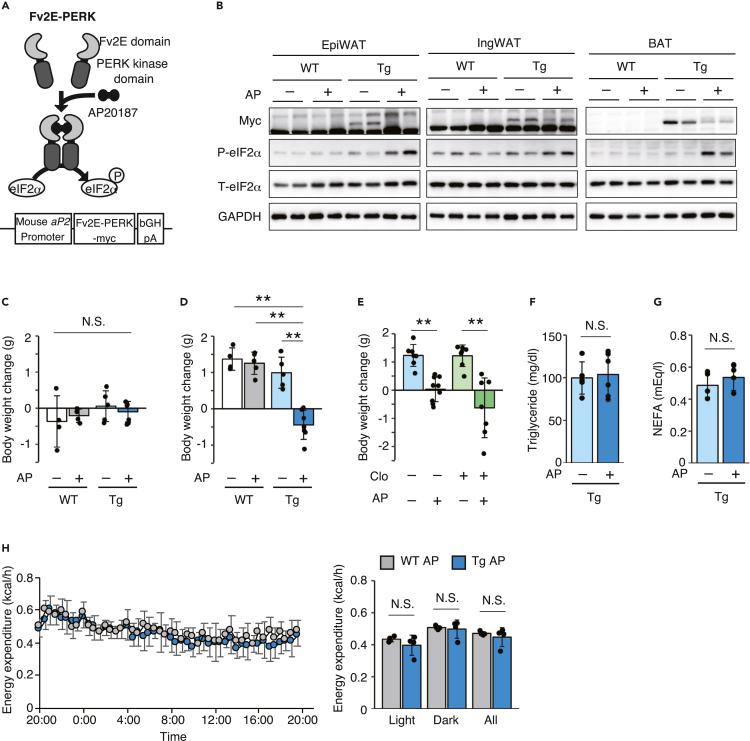
Acute phosphorylation of eIF2α in the adipose tissue only suppresses the increase in BW of mice fed the HFD (A) Scheme showing the generation of Fv2E-PERK and transgenic mice. (B) Representative immunoblots for Myc (Fv2E-PERK), phospho-eIF2α, total eIF2α, and GAPDH in the epiWAT, ingWAT, and BAT of WT and Tg mice at 6 h after the intraperitoneal injection of the vehicle or the artificial ligand AP21087 (AP) at 0.1 mg/kg BW. (C) Changes in BW 24 h after the injection of the vehicle or AP in WT and Tg mice fed the NCD (WT vehicle: *n* = 4, WT AP: *n* = 4, Tg vehicle: *n* = 5, Tg AP: *n* = 7). (D) Changes in BW 24 h after the injection of the vehicle or AP in WT and Tg mice fed the HFD (WT vehicle: *n* = 4, WT AP: *n* = 5, Tg vehicle: *n* = 5, Tg AP: *n* = 7). (E) Changes in BW 24 h after the injection of the vehicle or AP in Tg mice that were pre-treated with control liposomes or clodronate liposomes and fed the HFD (control vehicle: *n* = 7, control AP: *n* = 9, clodronate vehicle: *n* = 7, clodronate AP: *n* = 7). (F) Plasma triglyceride levels measured 24 h after the vehicle or AP injection in WT and Tg mice fed the HFD (Tg vehicle: *n* = 5, Tg AP: *n* = 7). (G) Plasma NEFA levels measured 24 h after the vehicle or AP injection in WT and Tg mice fed the HFD (Tg vehicle: *n* = 5, Tg AP: *n* = 7). (H) Energy expenditure measured immediately and up to 24 h after the injection of AP in WT and Tg mice fed the HFD (WT AP: *n* = 4, Tg AP: *n* = 4). All data are presented as means ± SD. Unpaired two-tailed Student's t tests were used to analyze the data presented in F, G, and H. One-way ANOVA followed by Holm-Sidak multiple comparisons tests were used to analyze the data presented in C, D, and E. ∗∗*P < 0.01*.

The ISR is activated in the adipose tissue of obese mice by lipotoxic stress and ER stress. Loss-of-function studies using tissue-specific KO mice of each ISR kinase have a limitation in determining the metabolic role of the ISR signaling pathway, because unresolved stress induced by ISR dysfunction might affect the pathophysiology of obesity and type 2 diabetes. We took advantage of the Fv2E-PERK chimeric protein, a fusion protein consisting of the eIF2α kinase domain of PERK and an artificial dimerization domain (Fv2E) that enables the specific and temporal activation of the ISR upon addition of the synthetic dimerizing molecule AP20187 (AP) without causing any stress and activation of other related signaling pathways, such as the unfolded protein response, to overcome this limitation (Lu et al., 2004). We generated Tg mice that expressed Fv2E-PERK in adipose tissue using the mouse aP2 (*Fabp4*) promoter cassette to assess the role of ISR signaling in adipose tissue (Figure 1A). Fv2E-PERK was expressed in the epididymal white adipose tissue (WAT), inguinal WAT, and BAT of the Tg mice, and an AP injection induced eIF2α phosphorylation in the epididymal WAT, inguinal WAT, and BAT (Figure 1B). As the aP2 promoter is known to be active in monocytes/macrophages and adipocytes, Fv2E-PERK expression was observed in the spleen and thioglycolate-induced peritoneal macrophages (Figures S1A and S1B), and the expression of the known ISR target genes *Ddit3* and *Ppp1r15a* was also induced by the AP treatment in peritoneal macrophages from Tg mice (Figure S1C).

We first monitored changes in body weight (BW) in WT and Tg mice after a single administration of AP during normal chow diet (NCD) and HFD feeding to explore the metabolic effects of ISR activation on the adipose tissue. Before administration, the BW of Tg mice was comparable with that of WT mice (Figure S2A). Significant differences were not observed among vehicle-injected WT, AP-injected WT, vehicle-injected Tg, and AP-injected Tg mice fed the NCD (Figure 1C). After changing to the HFD from the NCD, the BW of untreated WT mice was significantly increased within 24 h (data not shown). The administration of vehicle or AP to WT mice and vehicle to Tg mice increased the BW 24 h after the shift to the HFD, but the administration of AP to Tg mice suppressed the increase in BW (Figure 1D). Based on these results, ISR activation, but not the synthetic dimerizing molecule AP, suppressed the BW gain induced by the consumption of the HFD. We depleted macrophages in the Tg mice by injecting liposome-encapsulated clodronate to determine whether adipocytes or macrophages were responsible for the phenotype. Macrophage depletion was confirmed by the absence of F4/80-positive cells in the spleen (Figure S2B). The suppression of BW gain by AP in HFD-fed Tg mice was also observed after the clodronate liposome treatment, indicating that adipocytes, but not macrophages, were responsible for this phenotype (Figure 1E).

Because metabolism and energy expenditure in adipocytes affect BW regulation, we first assessed glucose and lipid metabolism in the AP-treated Tg mice. However, significant differences in blood glucose and plasma triglyceride and free fatty acid concentrations were not observed between vehicle- and AP-treated Tg mice fed the HFD (Figures S2D, 1F, and 1G). We next monitored the energy expenditure of the AP-treated Tg mice. AP administration to Tg mice did not affect energy expenditure assessed using an open circuit indirect calorimeter or physical activity assessed using a mouse movement analysis system (Figures 1H and S2C). Thus, ISR activation in adipocytes inhibited BW gain only under HFD feeding conditions, and the metabolic function of adipocytes was not related to this phenotype.

### Activation of the ISR in adipose tissue suppresses the intake of the HFD

**Figure 2:**
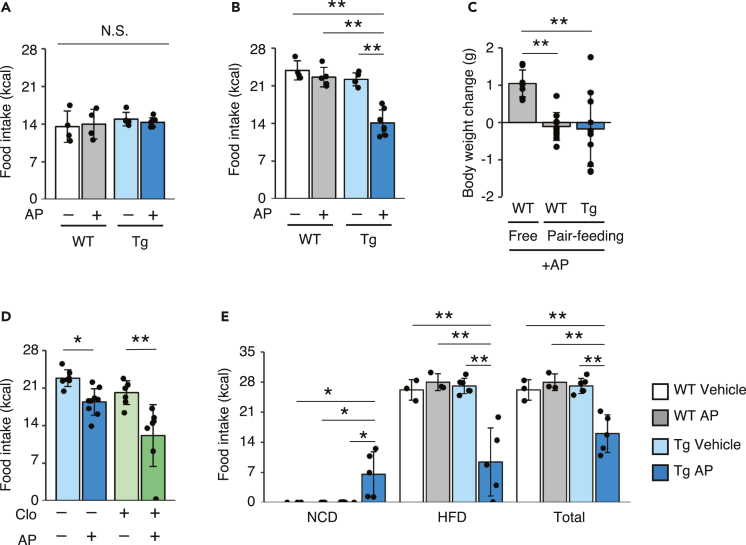
Acute phosphorylation of eIF2α in the adipose tissue suppresses the intake of the HFD (A) Food intake by vehicle- or AP-injected WT and Tg mice fed the NCD was measured for 24 h (WT vehicle: *n* = 4, WT AP: *n* = 4, Tg vehicle: *n* = 5, Tg AP: *n* = 7). (B) Food intake by vehicle- or AP-injected WT and Tg mice fed the HFD was measured for 24 h (WT vehicle: *n* = 4, WT AP: *n* = 5, Tg vehicle: *n* = 5, Tg AP: *n* = 7). (C) Changes in BW 24 h after the AP injection in free feeding and pair feeding WT mice and free feeding Tg mice (each group: n = 9). (D) Food intake by vehicle- or AP-injected Tg mice that had been pre-treated with control liposomes or clodronate liposomes and fed the HFD was measured for 24 h (control vehicle: *n* = 7, control AP: *n* = 9, clodronate vehicle: *n* = 7, clodronate AP: *n* = 7). (E) Food intake measured for 24 h during the two-diet choice (NCD and HFD) experiment using vehicle- or AP-injected WT and Tg mice (WT vehicle: *n* = 3, WT AP: *n* = 3, Tg vehicle: *n* = 6, Tg AP: *n* = 5). All data are presented as means ± SD. One-way ANOVA followed by Holm-Sidak multiple comparisons tests were used to analyze the data presented in (A–E). ∗*P < 0.05* and ∗∗*P < 0.01*.

When we monitored the food intake of the Tg mice after AP administration, we unexpectedly observed the suppression of food intake in AP-treated Tg mice fed the HFD, but not in mice fed the NCD (Figures 2A and 2B). The inhibition of BW gain and food intake was also observed in female Tg mice (Figures S3A and S3B). We monitored the change in BW under the conditions of “pair-feeding” in force-fed WT mice compared with AP-treated Tg mice provided with food *ad libitum* to confirm that the suppression of food intake was responsible for the reduction in BW gain induced by adipose-specific ISR activation. The BW gain of pair-fed WT mice was significantly lower than that of mice fed *ad libitum*, and no significant difference was observed compared with AP-treated Tg mice (Figure 2C). Consistent with the lack of involvement of macrophages in the HFD-specific BW loss in AP-treated Tg mice, the AP-induced suppression of food intake in HFD-fed Tg mice was also observed after the clodronate liposome treatment (Figure 2D). Based on these results, ISR activation in adipocytes elicited a suppression of food intake, specifically the HFD. When mice were provided free access to the NCD and HFD, WT mice treated with vehicle or AP and Tg mice treated with vehicle only consumed the HFD and did not eat any of the NCD. In contrast, Tg mice administered AP consumed the NCD instead of the HFD, suggesting that AP-treated mice tended to avoid the HFD (Figure 2E). The decreased appetite for the HFD in Tg mice treated with AP was consistent over another HFD purchased from a different manufacturer (Figure S3C). Thus, the HFD-specific BW loss was due to a specific aversion to the HFD triggered by ISR activation in adipocytes.

### Activation of the ISR in the adipose tissue of obese mice improves obesity by suppressing food intake

**Figure 3:**
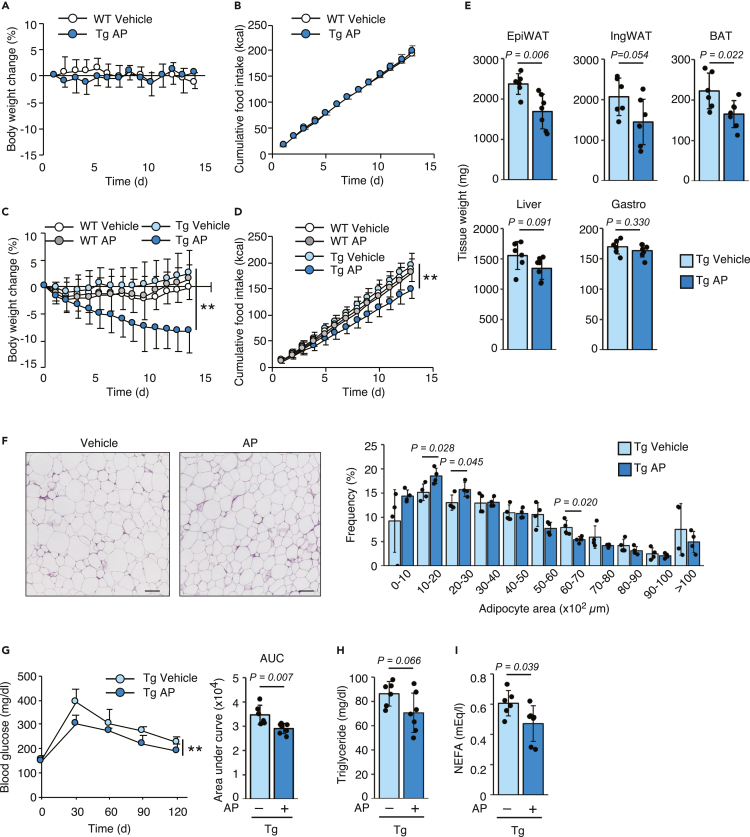
Chronic phosphorylation of eIF2α in adipose tissues of obese mice decreases the fat mass. WT and Tg mice were fed the NCD or HFD for 8 weeks beginning at 4 weeks old and were intraperitoneally injected with the vehicle or 0.1 mg/kg AP daily (A) Changes in the BW of vehicle-injected WT or AP-injected Tg mice fed the NCD (WT vehicle: *n* = 5, Tg AP: *n* = 5). (B) Cumulative food intake of vehicle-injected WT or AP-injected Tg mice fed the NCD (WT vehicle: *n* = 5, Tg AP: *n* = 5). (C) Changes in the BW of vehicle- or AP-injected WT and Tg mice fed the HFD (WT vehicle: *n* = 7, WT AP: *n* = 7, Tg vehicle: *n* = 13, Tg AP: *n* = 15). (D) Cumulative food intake by vehicle- or AP-injected WT and Tg mice fed the HFD (WT vehicle: *n* = 7, WT AP: *n* = 7, Tg vehicle: *n* = 13, Tg AP: *n* = 15). (E) Tissue weight of vehicle- or AP-injected Tg mice fed the HFD (Tg vehicle: *n* = 6, Tg AP: *n* = 7). (F) H&E staining in epididymal WAT sections from vehicle- or AP-injected Tg mice fed the HFD (scale bar, 100 μm). The frequency distribution of adipocyte sizes was analyzed (Tg vehicle: *n* = 4, Tg AP: *n* = 4). (G) Results of the glucose tolerance test and area under the curve for vehicle- or AP-injected Tg mice fed the HFD (Tg vehicle: *n* = 6, Tg AP: *n* = 7). (H) Plasma triglyceride levels in vehicle- or AP-injected Tg mice fed the HFD (Tg vehicle: *n* = 6, Tg AP: *n* = 7). (I) Plasma NEFA levels in vehicle- or AP-injected Tg mice fed the HFD (Tg vehicle: *n* = 6, Tg AP: *n* = 7). All data are presented as means ± SD. Unpaired two-tailed Student's t tests were used to analyze the data presented in (E–G) (AUC), (H and I). Two-way ANOVAs were used to analyze the data presented in (A–D) (Tg vehicle and Tg AP), and (G)x (kinetic data). ∗∗*P < 0.01*.

Similar to results of a single treatment, when mice were fed the NCD, we did not observe any significant changes in the BW and food intake of Tg mice treated daily with AP compared with WT mice treated daily with vehicle (Figures 3A and 3B). We next examined whether adipose-specific chronic ISR activation improves obesity and diabetes. Obesity and diabetes were induced in WT and Tg mice by feeding them the HFD, and no difference in BW was observed between the groups prior to the AP treatment (Figure S4A). A 2-week daily treatment with AP gradually decreased the BW up to 10% from the beginning of the experiments, but only in Tg mice (Figure 3C). Consistent with the decreased BW, the intake of the HFD by AP-treated Tg mice was also suppressed compared with the other groups (Figure 3D). Since the AP treatment did not produce any differences in WT mice compared with the vehicle, these effects resulted from ISR activation in adipocytes, but not side effects of the synthetic dimerizing molecule AP. The decreased BW was mainly due to fat loss in the adipose tissue (Figure 3E). HE staining revealed a significantly decreased size of adipocytes in the epididymal WAT of Tg AP mice (Figures 3F and S4B). Perhaps owing to the short duration of the treatment period, the difference in adipocyte size between AP-treated Tg mice and vehicle-treated Tg mice did not reach significance in the inguinal WAT and BAT (data not shown). Fewer lipid droplets were observed in the liver of AP-treated Tg mice than in vehicle-treated Tg mice (Figure S4B).

We next analyzed the effect of ISR activation on glucose and lipid metabolism. Glucose tolerance was improved after the AP treatment in Tg mice compared with the vehicle treatment (Figure 3G). Plasma concentrations of triglyceride and NEFA were decreased in AP-treated Tg mice (Figures 3H and 3I). We investigated the expression of metabolic regulatory genes in adipose tissue to exclude the possibility that ISR activation in adipose tissue reduces the fat mass by regulating the transcription of metabolism-related genes. AP-dependent ISR activation was confirmed by the mRNA expression of the well-known ISR target *Ddit3* in all adipose tissues (Figures S4C–S4E). In epididymal WAT, the levels of the *Fasn* and *Pparg* mRNAs, which are related to lipid metabolism, were lower in AP-treated Tg mice than in vehicle-treated Tg mice (Figure S4C). Only *Ucp1* expression was upregulated in the inguinal WAT of Tg mice (Figure S4D). Moreover, the mRNA levels of most genes related to thermogenesis, fatty acid oxidation, and lipid synthesis were decreased in the BAT of AP-treated Tg mice (Figure S4E). Thus, (1) ISR activation did not affect or only slightly altered fatty acid metabolism in the WAT and (2) ISR activation suppressed BAT activity (including thermogenesis and fatty acid metabolism) and might compensate for the induction of browning in inguinal WAT to maintain body temperature. Based on these results, the ISR-induced metabolic changes in adipose tissue were not the main contributor to the fat mass loss.

### ISR activation increased the expression of the *Gdf15* mRNA through the transcription factors ATF4 and DDIT3

**Figure 4:**
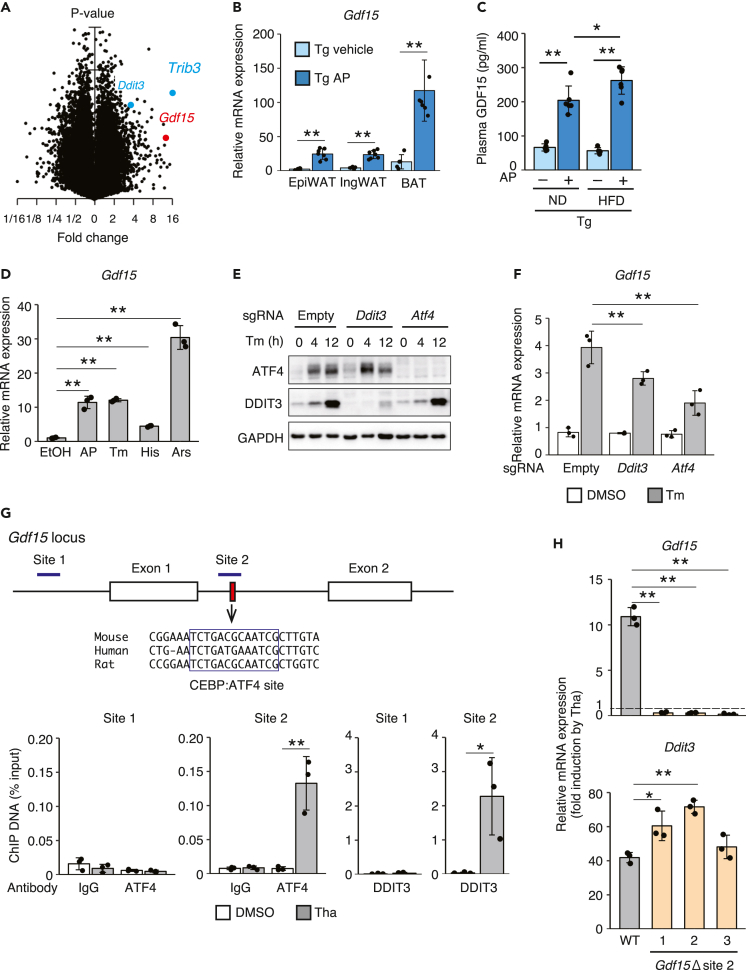
The secretion of the metabolic hormone GDF15 is induced by the phosphorylated eIF2α-ATF4-DDIT3 axis as an adipokine (A) Volcano plot comparing mRNA expression in the BAT of vehicle- or 0.05 mg/kg AP-injected Tg mice (n = 2). Mice were sacrificed at 8 weeks 12 h after the injection. Blue dots show the known targets of eIF2α phosphorylation *Ddit3* and *Trib3*. The red dot shows *Gdf15*. (B) RT-qPCR analysis of *Gdf15* mRNA expression in the BAT, ingWAT, and epiWAT of vehicle- or AP-treated Tg mice 24 h after the injection (Tg vehicle: *n* = 5, Tg AP: *n* = 7). (C) Plasma GDF15 concentrations in vehicle- or AP- treated Tg mice 24 h after the injection (Tg vehicle: *n* = 5, Tg AP: *n* = 7). (D) RT-qPCR analysis of *Gdf15* mRNA expression 12 h after treatment with AP, tunicamycin (Tm), histidinol (His), and arsenite (Ars) in 3T3L1 adipocytes stably expressing Fv2E-PERK (*n* = 3). (E) Representative immunoblots of ATF4, DDIT3, and GAPDH in 3T3L1 adipocytes stably expressing Cas9 and sgRNA against *Atf4* or *Ddit3* at 4 or 12 h after treatment with Tm. (F) RT-qPCR analysis of *Gdf15* mRNA expression in 3T3L1 adipocytes stably expressing Cas9 and sgRNAs against *Atf4* or *Ddit3* at 12 h after treatment with Tm (*n* = 3). (G) ChIP analysis of the ATF4 and DDIT3 occupancy of the *Gdf15* locus in MEFs 8 h after the thapsigargin (Tha) treatment (*n* = 3). (H) RT-qPCR analysis of *Gdf15* and *Ddit3* mRNA expression in MEFs lacking the ATF4/DDIT3-binding site at 10 h after treatment with Tha (*n* = 3). All data are presented as means ± SD. Unpaired two-tailed Student's t tests were used to analyze the data presented in (B and G). One-way ANOVA followed by Holm-Sidak multiple comparisons tests were used to analyze the data presented in (C, D, F, and H). ∗*P < 0.05* and ∗∗*P < 0.01*.

As described above, decreased intake of the HFD was responsible for the anti-obesity effect of adipose-specific ISR activation. Adipocytes secrete many hormones known as “adipokines” to regulate metabolism throughout the body. We compared the mRNA expression profile in the BAT of AP-treated Tg mice with vehicle-treated mice to identify the ISR mediator between the adipose tissue and brain that regulates food intake. The known ISR-related genes *Ddit3* and *Trib3* were upregulated by the AP treatment, indicating that this experiment worked well (Figure 4A and Table S2). The expression of leptin, a major adipokine that decreases food intake, was not changed by ISR activation in these mice (Table S2). The gene exhibiting the most prominent differential expression was *Gdf15* (Figure 4A). According to compelling evidence, GDF15 functions as an anti-obesity hormone by decreasing food intake (Emmerson et al., 2017; Hsu et al., 2017; Mullican et al., 2017; Yang et al., 2017). Therefore, we hypothesized that GDF15 might be the ISR mediator regulating food intake. The RT-qPCR analysis confirmed that the level of the *Gdf15* mRNA was significantly increased by AP in the epididymal WAT, inguinal WAT, and BAT (Figure 4B). Since the hypothalamus in the brain controls appetite, we examined Gdf15 expression in the hypothalamus. The expression level of Gdf15 mRNA in the hypothalamus of AP-treated Tg mice was similar to that of WT mice, in accordance with the lack of Fv2E-PERK expression (Figure S5A). Consistent with the high mRNA expression, the plasma concentration of GDF15 was increased in AP-treated Tg mice compared with vehicle-treated Tg mice (Figure 4C). We monitored the levels of the *Gdf15* mRNA in differentiated 3T3L1 adipocytes expressing Fv2E-PERK to validate that ISR activation directly induced the expression of the *Gdf15* mRNA. AP or other ISR inducers, including tunicamycin, histidinol, and arsenite, induced the expression of the *Gdf15* mRNA (Figure 4D).

The mitochondrial unfolded protein response was reported to induce *Gdf15* expression in skeletal muscle through the transcription factor DDIT3, but not ATF4 (Chung et al., 2017). Therefore, we examined whether ATF4 or DDIT3 mediates the ISR-induced upregulation of the *Gdf15* mRNA using *Atf4* and *Ddit3* knockdown 3T3L1 cells produced using CRISPR-Cas9-mediated genome editing. The successful knockdown of the ATF4 and DDIT3 proteins was accomplished in a pool of 3T3L1 cells transduced with the *Atf4* and *Ddit3* gRNA and Cas9 (Figure 4E). The expression of the *Gdf15* mRNA induced by tunicamycin was reduced in both *Atf4* or *Ddit3* knockdown 3T3L1 cells (Figure 4F). Since *Atf4* knockdown efficiently inhibited *Gdf15* expression to a greater extent than *Ddit3* knockdown, we assumed the direct regulation by ATF4 in addition to DDIT3. An *in silico* analysis allowed us to identify the novel sites containing the CEBP:ATF4-binding consensus sequence, which were conserved among mouse, human, and rat and located between exons 1 and 2 (Figure 4G). Therefore, chromatin immunoprecipitation (ChIP) analyses were performed to confirm that the newly identified site binds ATF4 and DDIT3. Both ATF4 and DDIT3 antibodies successfully detected the binding of ATF4 and DDIT3 to their previously reported target sites in the *Fgf21* and the *Ppp1r15a* promoters, respectively (Figures S5A and S5B). ATF4 and DDIT3 bound to our identified target site in the intron (site 2) but not the previously reported site in the promoter region (site1) of the *Gdf15* gene (Figure 4G). We re-analyzed the previously reported ChIP-seq data using different anti-ATF4 antibodies, and the results confirmed that ATF4 bound to this site in human and mouse cells after stimulation with ISR inducers (Figure S5C). We established three individual clones of MEF cells deficient for the CEBP:ATF4-binding site using CRISPR-Cas9-mediated genome editing to confirm the importance of the identified binding site in the *Gdf15* sequence in the regulation mediated by ISR (Figure S5D). All clones exhibited a complete loss of induction of *Gdf15* mRNA expression after treatment with the ISR inducer, although *Ddit3* expression was similar to the parental cells (Figure 4H). Furthermore, the cells containing the deleted CEBP:ATF4-binding site retained the ability to induce the expression of *Gdf15* mRNA after treatment with the NSAID indomethacin, which is known to induce the expression of the *Gdf15* mRNA via transcription factor EGFR1 (Figure S5E). *In vivo*, *Ddit3* KO mice showed slight but significant increases in BW and food intake compared with WT mice fed the HFD (Figures S6A and S6B). Although plasma GDF15 concentrations were reduced in *Ddit3* KO mice, the expression of the *Gdf15* mRNA tended to be lower but not significantly decreased in the adipose tissue, suggesting that both DDIT3 and ATF4 were likely required for GDF15 regulation in adipocytes (Figures S6C and S6D). Based on these data, ATF4 and DDIT3 cooperatively regulated Gdf15 expression triggered by ISR activation.

### The GDF15-GFRAL axis is responsible for the ISR-mediated control of food intake *in vivo*

**Figure 5:**
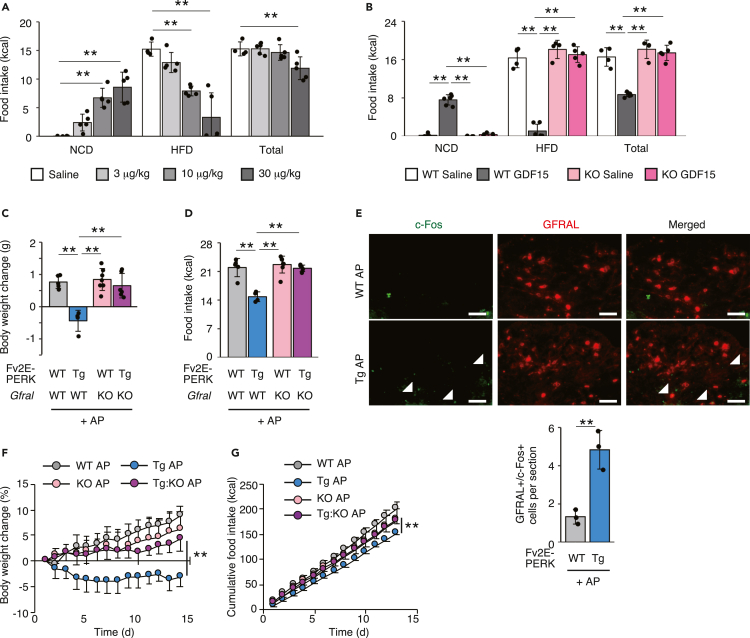
GFRAL is responsible for inhibiting the intake of the HFD through a mechanism mediated by phosphorylated eIF2α and GDF15 (A) Food intake measured for 12 h during the two-diet choice experiment (NCD and HFD) in WT mice treated with the indicated doses of GDF15 (0: *n* = 4, 0.003: *n* = 5, 0.01: *n* = 5, 0.03: *n* = 5). (B) Food intake measured for 12 h during the two-diet (NCD and HFD) choice experiment in WT or *Gfral*-deficient (KO) mice treated with 0.03 mg/kg GDF15 (WT saline: *n* = 4, WT GDF15: *n* = 5, KO saline: *n* = 4, KO GDF15: *n* = 5). (C) Changes in BW 24 h after the AP injection in WT, Tg, *Gfral* KO, and Tg:*Gfral* KO mice fed the HFD (WT AP: *n* = 5, Tg AP: *n* = 4, KO AP: *n* = 7, Tg:KO AP: *n* = 6). (D) Food intake measured for 24 h in AP-injected WT, Tg, *Gfral* KO, and Tg:*Gfral* KO mice fed the HFD (WT AP: *n* = 5, Tg AP: *n* = 4, KO AP: *n* = 7, Tg:KO AP: *n* = 6). (E) Representative photograph of area postrema immunostained for the c-Fos and GFRAL from WT and Tg mice treated with AP after 24 h (scale bar, 50 μm) (WT AP: *n* = 3, Tg AP: *n* = 3). Arrow heads show c-Fos and GFRAL double-positive cells. The graph below shows quantification of c-Fos and GFRAL double-positive cells in section. (F) Changes in BW of the AP-injected WT, Tg, *Gfral* KO, and Tg:*Gfral* KO mice fed the HFD (WT AP: *n* = 4, WT AP: *n* = 5, KO AP: *n* = 5, Tg:KO AP: *n* = 6). (G) Cumulative food intake of AP-injected WT, Tg, *Gfral* KO, and Tg:*Gfral* KO mice fed the HFD (WT AP: *n* = 4, WT AP: *n* = 5, KO AP: *n* = 5, Tg:KO AP: *n* = 6). All data are presented as means ± SD. Unpaired two-tailed Student's t tests were used to analyze the data presented in (E). One-way ANOVA followed by Holm-Sidak multiple comparisons tests were used to analyze the data presented in (A–D). Two-way ANOVAs were used to analyze the data presented in (F and G) (Tg AP and Tg:KO AP). ∗∗*P < 0.01*.

Researchers have not determined whether the food composition modulates the effects of GDF15 on suppressing appetite and weight gain. Plasma human GDF15 concentrations were detected 1 h after subcutaneous injection of rhGDF15 (Figure S7A). The administration of 0.1 mg/kg rhGDF15 to mice fed the HFD completely inhibited food intake and reduced BW (Figures S7B and S7C). When 0.03 mg/kg rhGDF15 was injected, reduction of food intake and BW were more apparent in the HFD-fed mice than in the NCD-fed mice (Figures S7D and S7E). We observed a reduction in HFD intake and increase in NCD intake after treatment with a low amount of GDF15 when mice had free access to the NCD and HFD (Figure 5A). Then, we established GDF15 receptor *Gfral* KO mice using CRISPR-Cas9-mediated genome editing (Figure S7F). The GFRAL protein was expressed in the area postrema of the hindbrain of WT mice and was depleted in that of our *Gfral* KO mice (Figure S7G). The administration of GDF15 to *Gfral* KO mice completely abolished the specific aversion to the HFD observed in WT mice (Figure 5B). Thus, GDF15 controlled the preference for the HFD via GFRAL. *Gfral* KO mice were crossed with our Tg mice to confirm that GDF15 mediates the suppression of the appetite for the HFD upon ISR activation in adipocytes. The BWs of WT, Tg, *Gfral* KO, and Tg:*Gfral* KO mice were similar (Figure S7H). Although plasma GDF15 concentrations were increase in Tg:*Gfral* KO mice, similar to Tg mice, the GFRAL deficiency in Tg mice completely abolished the suppression of BW gain and food intake (Figures 5C, 5D, and S7I). Activation of GFRAL-positive neurons was evaluated by the known activation marker c-Fos. GFRAL and c-Fos double-positive neurons were increased in the area postrema of AP-treated Tg mice compared with that of WT mice treated with AP (Figure 5E). Furthermore, ISR activation in Tg:*Gfral* KO mice after the development of obesity did not improve obesity (Figure 5F). Consistent with this finding, the reduction in food intake observed in Tg mice was also reversed in Tg:*Gfral* KO mice (Figure 5G). Thus, ISR activation in adipocytes controlled food intake through the GDF15-GFRAL axis.

### 10(E), 12(Z)-Octadecadienoic acid-induced GDF15 expression in adipocytes depends on the ISR kinase PERK and suppresses HFD intake

**Figure 6:**
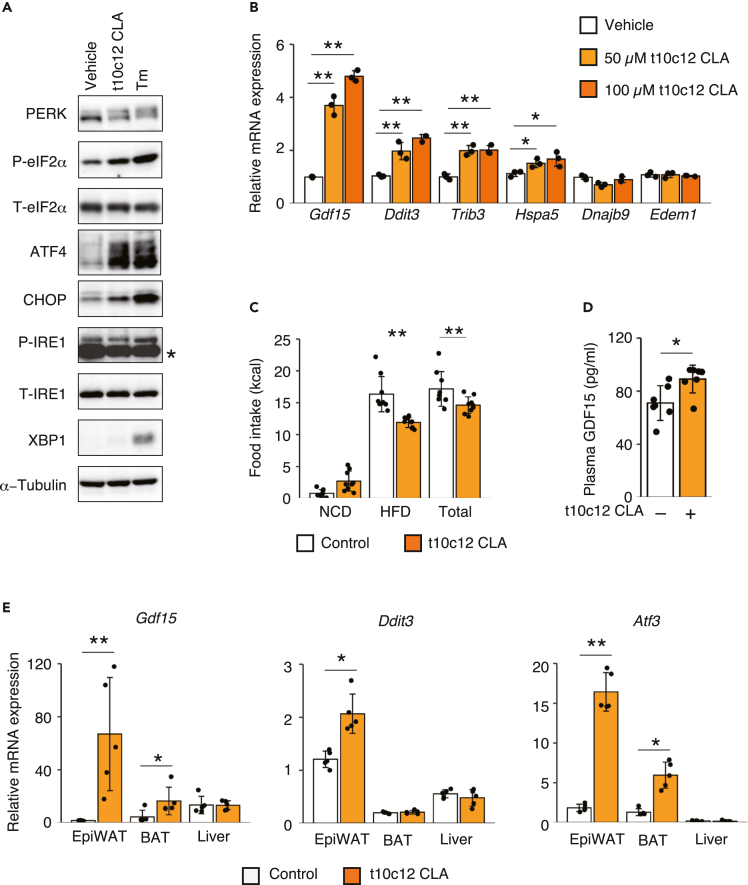
10(E),12(Z)-Octadecadienoic acid activates the ISR in adipocytes and suppresses HFD intake (A) Representative immunoblots for PERK, phospho-eIF2α, total eIF2α, ATF4, CHOP, phospho-IRE1, total IRE1, XBP1, and α-tubulin in 3T3L1 adipocytes at 12 h after treatment with 100 μM t10c12 CLA or Tm. The asterisk indicates a nonspecific band. (B) RT-qPCR analysis of *Gdf15, Ddit3, Trib3, Hspa5, Dnajb9,* and *Edem1* mRNA in 3T3L1 adipocytes at 12 h after treatment with 50 or 100 μM t10c12 CLA (*n* = 3). (C) Food intake measured for 24 h in WT mice fed t10c12 CLA or olive oil (control) (*n* = 8). (D) Plasma GDF15 concentrations in mice fed 1% t10c12 CLA or olive oil (control) (*n* = 7). (E) RT-qPCR analysis of *Gdf15, Ddit3,* and *Atf3* mRNA in the epiWAT, BAT, and livers of mice fed t10c12 CLA or olive oil (control) (*n* = 5). All data are presented as the means ± SDs. Unpaired two-tailed Student's t tests were used to analyze the data. ∗*P < 0.05* and ∗∗*P < 0.01*.

We searched for conditions that induce GDF15 expression and activate the ISR using the curated expression database Genevestigator to pursue physiological/pharmacological relevance of our findings. Notably, 10(E), 12(Z)-octadecadienoic acid (t10c12 CLA), one of main conjugated linoleic acids present in foods, increased *Gdf15* expression to the greatest extent in adipocytes. Indeed, LaRosa et al. reported that t10c12 CLA activated the ISR in adipocytes (LaRosa et al., 2007). We confirmed that t10c12 CLA increases PERK phosphorylation, eIF2α phosphorylation, and ATF4 and CHOP expression in 3T3L1 adipocytes (Figure 6A). Although weak induction of *Hspa5* expression was observed, t10c12 CLA did not increase IRE1 phosphorylation, XBP1 expression, and the levels of other ER stress markers, such as *Dnajb9* and *Edem1*, indicating that t10c12 CLA did not induce substantial ER stress (Figures 6A and 6B). Activation of the ISR by the t10c12 CLA treatment was further confirmed by the result showing that treatment with the ISR inhibitor ISRIB reduced the induction of *Gdf15* and *Atf4* expression (Figures S8B and S8C). Interestingly, the induction of *Gdf15* and *Ddit3* expression was observed in adipocytes, such as differentiated 3T3L1 adipocytes and brown adipocytes but was absent or detected at very low levels in non-adipocyte cells, such as undifferentiated 3T3L1 cells, C2C12 myoblasts, and RAW264.7 macrophages, indicating that the adipose tissue is a target of t10c12 CLA for GDF15 induction (Figure S8A).

Next, we examined the effects of t10c12 CLA *in vivo*. Mice were first fed a t10c12 CLA-containing diet or control olive oil-containing diet and then provided free access to the NCD and HFD. The t10c12 CLA-treated mice consumed less of the HFD and more of the NCD than the olive oil-treated mice, which was accompanied by an increase in the plasma GDF15 level (Figures 6C and 6D). Further support for our hypothesis was obtained from the results of cell experiments, the expression of the *Gdf15* mRNA and ISR-related genes *Ddit3* and *Atf3* was increased in the epididymal WAT and BAT, but not the liver, indicating an important role for the ISR-GDF15 axis, at least from the pharmacological perspective, and potentially represents a therapeutic target for type 2 diabetes and obesity (Figure 6E).

## Discussion

The ISR exerts a cytoprotective effect on various types of stress and is implicated in the regulation of whole-body metabolism. In this study, adipocyte-specific ISR activation protects mice from diet-induced obesity and type 2 diabetes by inhibiting HFD intake. The comprehensive transcriptome analysis of adipose tissue revealed that the expression of adipocyte-derived *Gdf15* is directly induced by the ISR via the transcription factors ATF4 and DDIT3 and suppresses the appetite for the HFD through its receptor GFRAL. Furthermore, pharmacological activation of the ISR by 10E, 12Z-octadecadienoic acid also induced GDF15 expression in adipocyte and decreased HFD intake *in vivo*. These findings highlight the non-cell-autonomous metabolic regulation of the adipocyte-specific ISR.

ISR is now emerging as a regulator of whole-body homeostasis in a non-cell-autonomous manner. Various stresses occur not only at the cellular level but also at the whole-organism level under physiological and pathological conditions. Therefore, the observation that higher organisms have developed a mechanism to cope with stress, even at the whole-organism level, via interorgan communication is reasonable. In the context of metabolic homeostasis, we and other researchers have reported that the ISR in skeletal muscle (Kim et al., 2013; Miyake et al., 2016) or liver (Kang et al., 2021; Xu et al., 2018) non-cell-autonomously regulates metabolism in other tissues via FGF21. In the current study, adipose-derived GDF15 induced by the ISR regulates the brain appetite systems via the GDF15 receptor GFRAL. A previous study showed that GDF15 is increased in adipocytes by mitochondrial stress, which is known to activate ISR (Choi et al., 2020). Thus, the GDF15-GFRAL axis is the main mediator that controls whole-body metabolism through the ISR in adipocytes. As a peripheral hormone, FGF21, another mediator of the ISR, has anti-obesity effects (Kim et al., 2013; Xu et al., 2018). Our current finding is consistent with the results of the previous FGF21 study, suggesting that ISR signaling may be improving whole-body metabolism of metabolic disorder in a non-cell-autonomous manner.

GDF15 is expressed in various tissues, including the liver, muscle, kidney, and adipose tissue (Ding et al., 2009). For example, GDF15 expression is induced in osteocytes via the transcription factor HIF-1α under severe hypoxic conditions in bone (Hinoi et al., 2012). In macrophages or tumor cells, the transcription factor STAT6 (Jung et al., 2018) or NF-kB (Ratnam et al., 2017) is reported to be involved in inducing GDF15 expression. Adipocytes and hepatocytes secrete GDF15 upon defect of oxidative phosphorylation through the adaptive mitochondrial stress response (Choi et al., 2020; Kang et al., 2021). Thus, GDF15 secretion generally increases in response to cellular stress or injury and stress-responsive transcription factors are suggested to induce the expression of the *Gdf15* mRNA in a context-dependent manner. In this study, DDIT3, which was previously implicated in the regulation of *Gdf15*, and ATF4 directly bind a previously unidentified enhancer located in the intron between exons 1 and 2 of *Gdf15*. ATF4 and DDIT3 interact and bind to specific sites to control the expression of target genes (Han et al., 2013). Based on the ChIP results, DDIT3 and ATF4 also interacted with the binding site in the *Gdf15* gene and maximized the induction of GDF15 expression. ISR-ATF4/DDIT3-dependent GDF15 regulation may be important in many different diseases and the therapeutic effects of drugs related to anorexia, cancer, and metabolic disorders.

Unexpectedly, the activation of the GDF15-GFRAL axis by ISR or exogenous administration of GDF15 preferentially inhibited HFD intake. GDF15 probably controls food preference through direct action in the central nervous system because depletion of GFRAL, whose expression is restricted to the brainstem, results in a loss of the effects of GDF15. Previous studies examining the suppression of both NCD and HFD intake did not mention the difference between NCD and HFD intake induced by GDF15 administration (Hsu et al., 2017). Moreover, according to a recent paper, activation of the GDF15-GFRAL axis causes emesis and nausea (Patel et al., 2019). Since the amount of exogenous or induced GDF15 secretion in these studies was much higher than in our study, the effect of GDF15 might be too strong to observe the suppression of the appetite for the HFD, because the suppression of the appetite for the NCD masked the negative alliesthesia for the HFD. Our results provide a new perspective of the ability of a low concentration of GDF15 to control food preferences, presumably under physiological conditions. The types of chemical cues, such as taste or odor, involved in GDF15-induced alimentary alliesthesia and its mechanism of action in the control of food intake must be determined in future studies.

The induction of GDF15 secretion mediated by ISR activation reduced food intake and BW, despite the increased GDF15 concentration in the blood of obese mice, suggesting that central neurons may display less resistance to GDF15 than leptin. Given the importance of GDF15, pharmacological modulators of GDF15 secretion could be therapeutically exploited, in addition to GDF15 administration or GFRAL agonists (Xiong et al., 2017). In recent years, several small compounds that modulate ISR signaling have been identified. For example, pharmacological activation of ISR signaling has been achieved either by activating eIF2α kinases or inhibiting eIF2α phosphatase. Salubrinal (Boyce et al., 2005), guanabenz (Tsaytler et al., 2011), or its derivatives sephin 1 (Das et al., 2015) and raphin 1 (Krzyzosiak et al., 2018) increase eIF2α phosphorylation by inactivating eIF2α phosphatases. In the present study, conjugated linoleic acid t10c12 CLA induced GDF15 expression in adipocytes and controlled the appetite for HFD via ISR. Although the detailed mechanism remains unknown, previous reports suggested that t10c12 CLA reduced body weight in obese mouse and human, respectively (Blankson et al., 2000; den Hartigh et al., 2018). In the present study, ISR/GDF15 in adipocytes was an important mediator of the effects of t10c12 CLA. A tissue-specific ISR enhancer such as t10c12 CLA might enable the moderate and long-term induction of GDF15 expression rather than a whole-body ISR inducer or exogenous GDF15/GFRAL agonist. Actually, the anti-diabetic drug metformin suppresses appetite by inducing GDF15 expression through a mechanism regulated by ISR in kidney and intestine or liver (Coll et al., 2020). Mice injected with metformin had increased concentrations of GDF15 in the blood and ate an NCD instead of an HFD under free access to both the NCD and HFD (Figures S9A and S9B). GDF15 has been proposed to be an anti-obesity drug. However, taking into account the ISR-induced anti-obesity effects of FGF21 in addition to GDF15, ISR enhancers represent attractive alternatives for appetite control in patients with metabolic disorders.

### Limitations of the study

ISR activation in adipocytes controls appetite through regulation of GDF15 expression and secretion. The experiments using t10c12 CLA indicated the pharmacological significance of this pathway. However, pathophysiological importance of ISR in adipocytes, for example, in obesity and diabetes, is not well demonstrated in this study. In addition, we also showed that GDF15 controlled the preference for the HFD via GDF15-GFRAL. It is required to reveal the mechanism of this change for clinical application.

## STAR★Methods

### Key resources table

**Table:** 

REAGENT or RESOURCE	SOURCE	IDENTIFIER
**Antibodies**		

Anti-Myc-tag	MBL	Cat# M192-3, RRID:AB_11160947
Anti-DDIT3	Proteintech	Cat# 15204-1-AP, RRID:AB_2292610
Anti-eIF2α	Cell Signaling Technology	Cat# 9722, RRID:AB_2230924
Anti-phospho-eIF2α (Ser51)	Cell Signaling Technology	Cat# 9721, RRID:AB_330951
Anti-ATF4	Cell Signaling Technology	Cat# 11815, RRID:AB_2616025
Anti-β-actin	MBL	Cat# M177-3, RRID:AB_10697039
Anti-GAPDH	MBL	Cat# M171-3, RRID:AB_10597731
Anti-F4/80	BioLegend	Cat# 123101, RRID:AB_893504
Anti-GFRAL	R&D Systems	Cat# AF5728 RRID:AB_2110592
Anti-c-Fos	Cell Signaling Technology	Cat# 2250 RRID:AB_2247211

**Chemicals, peptides, and recombinant proteins**		

AP21087 (B/B Homodimerizer)	Clontech	Cat# 635058
Human recombinant GDF15	Peprotech	Cat# 120-28C
Clodronate liposomes	Hygieia	Cat# 16003642
10E, 12Z-Octadecadienoic Acid	Cayman Chemical	Cat# 90145
10E, 12Z-Octadecadienoic Acid	Larodan Fine Chemicals	Cat# 10-1826-90
Insulin, Human, recombinant	WAKO	Cat# 099-06473
3-Isobutyl-1-methylxanthine	SIGMA ALDRICH	Cat# I-5879
Troglitazon	WAKO	Cat# 207-17601
Dexamethasone	WAKO	Cat# 041-18861
T3 (3,3',5-Triiodo-L-thyronine)	Nacalai Tesque	Cat# 35006-44
Rosiglitazone	WAKO	Cat# 180-02653
Indomethacin	Nacalai Tesque	Cat# 19233-51
Polyethylenimine	WAKO	Cat# 24765
Polybrene (Hexadimethrine bromide)	SIGMA ALDRICH	Cat# H9268
Puromycin	Goldbio	Cat# P-600-1
Tunicamycin	Calbiochem	Cat# 654380
Histidinol	TRC canada	Cat# H456300
Sodium arsenite	SIGMA ALDRICH	Cat# S7400
Bovine Serum Albumin Fatty acid-Free	Equitech-Bio	Cat# BAH66-0100
O.C.T. compound	Sakura Finetech	Cat# 4583
Hoechst 33258	Thermo Scientific	Cat# H3569
Protease Inhibitor Cocktail	Biotool	Cat# B14003
Phosphatase Inhibitor Cocktail	Biotool	Cat# B15002
EvaGreen	Biotium	Cat# 31000
Protein A-Sepharose	Invitrogen	Cat# 101042
Alt-R S.p. HiFi Cas9 Nuclease V3	IDT	Cat# 1081060

**Critical commercial assays**		

Triglyceride E Test	WAKO	Cat# 432-40201
Cholesterol E Test	WAKO	Cat# 439-17501
Non-esterified fatty acid E Test	WAKO	Cat# 279-75401
Mouse/Rat GDF-15 Quantikine ELISA Kit	R&D Systems	Cat# MGD150
Human GDF-15 Quantikine ELISA Kit	R&D Systems	Cat# DGD150
Immobilon Western Chemiluminescent HRP Substrate	Millipore	Cat# WBKLS0500
ReverTra Ace® qPCR RT Master Mix with gDNA Remover	TOYOBO	Cat# FSQ-301
AmpliTaq Gold 360 Master Mix	Thermo Scientific	Cat# 4398886
RNeasy MinElute Cleanup Kit	Qiagen	Cat# 74204
Agilent LowInput QuickAmp Labeling Kit	Agilent Technologies	Cat# 5190-2330
SurePrint G3 Mouse Gene Expression 8x60K Microarray Kit	Agilent Technologies	Cat# G4852A

**Deposited data**		

Microarray data of Tg mice	This paper	GSE149423

**Experimental models: Cell lines**		

3T3-L1	ATCC	CL-173, RRID:CVCL_0123
Immortalized brown preadipocytes	This study	N/A
RAW264.7	ECACC	91062702, RRID:CVCL_049
C2C12	ATCC	CRL-3419, RRID:CVCL_UR38
Mouse embryonic fibroblast	This study	N/A

**Experimental models: Organisms/strains**		

Mouse: *maP2-Fv2E-PER*K	This study	N/A
Mouse: *Gfral*^*−/−*^	This study	N/A
Mouse: *Ddit3*^*−/−*^	Oyadomari et al. (2001)	N/A
Mouse: WT: C57BL/6J	Charles River	027

**Oligonucleotides**		

Primers for qPCR, ChIP, see Table S1	This study	N/A
Mouse guides sequences for CRISPR/Cas9, see Table S1	This study	N/A

**Recombinant DNA**		

pCDF1 Lentivirus System	System Biosciences	Cat# CD110B-1
pCDF1-Fv2E-PERK	Miyake et al., 2016	N/A
lentiCRISPR v2	Addgene	Plasmid# 52961
pMD2.G	Addgene	Plasmid# 12259
psPAX2	Addgene	Plasmid# 12259
pSp-Cas9-2A-GFP	Addgene	Plasmid# 12260

**Software and algorithms**		

CRISPOR	Concordet and Haeussler, 2018	http://crispor.tefor.net/crispor.py
Genomatix Suite	Genomatix	N/A
ChIP-Atlas	Oki et al. (2018)	https://chip-atlas.org/
Integrative Genomics Viewer	Broad Institute	http://software.broadinstitute.org/software/igv/
GENEVESTIGATOR	Nebion	N/A
BZ-X analyzer	Keyence	N/A
R v3.5.2	RCore Team, R Foundation for Statistical Computing	https://www.r-project.org/

**Other**		

High Fat Diet (HFD; 60% kcal fat)	Research diets	Cat# D12492
High Fat Diet (HFD; 60% kcal fat)	Oriental Yeast	Cat# HFD-60

### Resource availability

#### Lead contact

Further information and requests for resources and reagents should be directed to and will be fulfilled by the Lead Contact, Masato Miyake (miyake@genome.tokushima-u.ac.jp).

#### Material availability

All plasmids and mice within this study were acquired as per the following method section details. Request for further information should be directed to the lead contact.

### Experimental model and subject details

#### Mice

A mouse aP2 (*Fabp4*) promoter DNA fragment (5.4 kb) was used to develop Tg mice that specifically express Fv2E-PERK in white and brown adipose tissues. Tg mice (Accession No. CDB0534T: http://www2.clst.riken.jp/arg/TG%20mutant%20mice%20list.html) were established in the C57/BL6N strain. Genotyping were performed by PCR using primer listed in S1 Table (product size: 201 bp). *Ddit3* knockout (KO) mice (Oyadomari et al., 2001) were backcrossed with C57BL/6J mice for at least 8 generations. The mice were fed a normal chow diet (NCD: Oriental Yeast Co.) containing 13.2% fat or a HFD containing 60% fat (Research Diets or Oriental Yeast Co.: only used in Figure S3A) and raised under specific pathogen-free conditions at the Institute of Advanced Medical Sciences of the University of Tokushima, Japan. Male mice were used for all experiment except for Figures S3A and S3B, which used female mice. For single treatments, mice (8 – 20 weeks old) were administered AP21087 (ARIAD Pharmaceuticals/Clontech) or vehicle (4% ethanol, 10% polyethylene glycol-400, and 1.75% Tween-20 in water) through an intraperitoneal (i.p.) injection according to manufacturer’s instructions at 8:00 AM – 11:00 AM in all experiments except for measurement of energy expenditure (8:00 PM). Clodronate liposomes (Hygieia) were intraperitoneally injected twice at 48 h and 24 h prior to AP20187 administration into 8 weeks old mice. Human recombinant GDF15 (Peprotech) was dissolved in saline and subcutaneously injected at 6:00 PM – 7:00 PM into 8 weeks old mice. For chronic treatments, mice were fed the NCD or the HFD for 8 weeks beginning at 4 weeks of age and then AP20187 was administered daily to these mice as described above up to 16 days. For the food choice tests, the mice (8 – 10 weeks old) were allowed free access to the NCD and the HFD in the cage. For the 10(E), 12(Z)-octadecadienoic acid (t10c12 CLA; Larodan Fine Chemical, 90% purity) treatment, mice (8 – 10 weeks old) were fed 1% t10c12 CLA or olive oil containing NCD for 4 days and orally gavaged with 50 μL of 40% t10c12 CLA-containing olive oil or olive oil alone. In all studies, mice were separated and acclimated at least 3 days before the experiment. The Animal Research Committee of the University of Tokushima and Institutional Animal Care and Use Committee (IACUC) of RIKEN Kobe Branch approved this study.

#### Cells

3T3L1 preadipocytes, immortalized brown preadipocytes, RAW264.7 cells, C2C12 cells, and mouse embryonic fibroblasts (MEFs) were cultured in Dulbecco’s Modified Eagle’s Medium (DMEM) supplemented with 10% fetal bovine serum (FBS). Confluent 3T3L1 preadipocytes were induced to differentiate in medium containing 10% FBS, 10 μg/ml insulin, 1 μM troglitazone, 0.5 mM isobutylmethylxanthine (IBMX), and 0.4 μg/ml dexamethasone for 2 d and subsequently cultured in DMEM containing 10% FBS and 5 μg/ml insulin for an additional 6 d. Confluent immortalized brown preadipocytes were induced to differentiate in medium containing 10% FBS, 5 μg/ml insulin, 1 nM T3, 0.5 μM rogiglitazone, 0.5 mM isobutylmethylxanthine (IBMX), 2 μg/ml dexamethasone, and 125 μM indomethacin for 2 d and subsequently cultured in DMEM containing 10% FBS, 5 μg/ml insulin, and 1 nM T3 for an additional 6 d. Confluent C2C12 cells were induced to differentiate in DMEM containing 2% horse serum for 4 d. 3T3L1 cells stably expressing Fv2E-PERK were established using the pCDF1 Lentivirus System (System Biosciences) as previously described (Miyake et al., 2016). 3T3L1 preadipocytes were infected with the lentivirus-containing supernatant in the presence of 8 μg/ml polybrene and were selected using 3 μg/ml puromycin. The ISR was induced by adding 2 μg/ml tunicamycin (Merck Millipore), 2 mM histidinol (Toronto Research Chemicals) and 50 μM sodium arsenite (Sigma-Aldrich) to the culture medium of differentiated 3T3L1 adipocytes. MEFs were stimulated with 0.2 μM thapsigargin (Cayman Chemical) or 100 mM indomethacin (Nacalai Tesque). The t10c12 CLA dissolved in EtOH (Cayman Chemical) was conjugated with fatty acid-free bovine serum albumin (Equitech-Bio).

### Method details

#### CRISPR/Cas9-mediated genome editing in cell lines and mouse zygotes

Genomic editing was performed using the CRISPR/Cas9 system as previously described (Sanjana et al., 2014). Briefly, small guide RNAs (sgRNAs) targeting the *Atf4*, *Ddit3*, and *Gdf15* (intron) genes were designed using online software (Concordet and Haeussler, 2018) (http://crispor.tefor.net/crispor.py). For *Atf4* and *Ddit3*, dsOligos were cloned into the lentiCRISPR v2 plasmid (52961; Addgene). Lentiviruses were generated in 293T cells by cotransfecting the cells with the lentiCRISPR plasmid, pMD2.G (addgene:12259) plasmid and psPAX2 (addgene:12260) plasmid using polyethylenimine (Polysciences), and supernatants were harvested after 48 h. 3T3L1 preadipocytes were infected with the lentivirus-containing supernatant in the presence of 8 μg/ml polybrene and selected with 3 μg/ml puromycin for at least 4 days beginning 24 h after the infection. Pooled cells were used in this study. For *Gdf15*, dsOligos were cloned into the pSp-Cas9-2A-GFP plasmid (48138; Addgene). Two plasmids containing oligos for different target sites were co-transfected into MEFs using the Neon Transfection System (Thermo Fisher Scientific). GFP-positive transfected cells were sorted using an S3 cell sorter (Bio-Rad). Clone cells in which the target sites were deleted were obtained using the limiting dilution method.

Deletion of Gfral in mouse zygotes was achieved by electroporating a Cas9–gRNA ribonucleoprotein complex mixture composed of two crRNAs targeting exon 5 and intron 5 of *Gfral*. Briefly, two synthetic crRNAs were first hybridized with tracrRNA and then assembled with recombinant Cas9 protein, using the Alt-R CRISPR-Cas9 System (IDT). One-cell zygotes were prepared by *in vitro* fertilization of C57BL/6J mouse oocytes and sperm. Genome Editor electroporator (BEX Co) and LF501PT1-5 platinum plate electrode (BEX Co) were used for electroporation. 30–40 zygotes were placed in a line in the electrode gap filled with 5 μl of Opti-MEM I containing RNP complex, and subjected to electroporation with 30V (3 msec ON + 97 msec OFF) for 7 times. After overnight culture, the resulting two-cell embryos were transferred into the oviducts of pseudo-pregnant MCH/ICR females. The obtained heterozygous mice expressing the deleted *Gfral* were crossed to each other to generate *Gfral* KO mice.

The deletion of target sites was determined through PCR amplification and confirmed by sequencing. All oligo sequences were listed in Table S1.

#### Physiological analysis

Energy expenditure was measured using indirect calorimetry after at least two days of acclimation using the Comprehensive Lab Animal Monitoring System (Columbus Instruments). Activity was measured using LOCOMO (MELQUEST). Food intake was measured at the indicated time points. For the glucose tolerance test, mice were fasted overnight (14 h) and orally gavaged with 1 g/kg glucose in PBS. Blood glucose levels were measured in blood collected from the tail vein using a OneTouch Ultra glucometer (LifeScan).

#### Histological analysis

Epididymal and inguinal white adipose tissue (WAT), brown adipose tissue (BAT) and the liver were fixed with 4% paraformaldehyde overnight. Paraffin sections (5 μm) were prepared and stained with hematoxylin and eosin (HE). Spleen were embedded in O.C.T. compound (Sakura Finetech) and frozen in a dry ice acetone bath. Frozen sections (10 μm) were fixed with acetone and stained with an anti-F4/80 antibody (Biolegend) and Alexa Fluor 488-conjugated donkey anti-rat IgG (H+L) (Thermo Scientific). Nuclei were stained with Hoechst 33258 (Thermo Scientific). Brain were obtained after transcardially perfused with 4% paraformaldehyde in 0.1 M phosphate buffer and post-fixed overnight at 4°C. The sampled were embedded in O.C.T. compound (Sakura Finetech) and frozen in a dry ice acetone bath. Brains were cut into 15 μm-thick coronal sections. Immunohistochemistry was performed using anti-GFRAL (R&D Systems) and anti-FOS (Cell signaling technology) for first antibody, and Alexa Fluor 488-conjugated donkey anti-rabbit IgG (H+L) and Alexa Fluor 594-conjugated donkey anti-sheep IgG (H+L) (Thermo Scientific) for second antibody, as previously described (Hsu et al., 2017). Photographs were taken and analyzed under a microscope with an imaging system (KEYENCE, #BZ-X700 or BZ-X800). Averages of 4 sections per animal were used to quantify the number of c-Fos and GFRAL labeled cells in the area postrema.

#### Biochemical analyses

The plasma triglyceride, cholesterol, non-esterified fatty acid (NEFA), mouse and human GDF15 concentrations were measured using the Triglyceride E-Test (Wako), Cholesterol E-Test (Wako), NEFA C- Test (Wako) and Mouse/Rat GDF15 Quantikine ELISA Kit (R&D Systems), Human GDF-15 Quantikine ELISA Kit (R&D Systems), respectively.

#### Immunoblot analysis

Lysates were prepared in RIPA buffer (50 mM Tris-HCl, 150 mM NaCl, 1% NP-40, 0.1% SDS, and 0.5% sodium deoxycholate) containing a protease inhibitor cocktail (Biotool) and phosphatase inhibitor cocktail (Biotool). Tissue homogenates were generated using a Polytron homogenizer (Kinematica). The protein concentrations of lysates were measured using the bicinchoninic acid method, and immunoblot analyses were performed as described previously (Miyake et al., 2016). The following antibodies were used: anti-Myc (MBL), anti-DDIT3 (Proteintech), anti-eIF2α (Cell Signaling Technology), anti-phospho-eIF2α (Ser51: Cell Signaling Technology), anti-ATF4 (Cell signaling technology), anti-β-actin (MBL), and anti-GAPDH (MBL). Bands were detected using Immobilon Western Chemiluminescent HRP Substrate (Millipore), and images were acquired using an EZ-Capture II Cooled CCD Camera System (ATTO Corp., Tokyo, Japan).

#### Analysis of mRNA expression

Total RNA was extracted from tissue or cell samples using AGPC methods and was used as a template for cDNA synthesis with the ReverTra Ace qPCR RT Master Mix and gDNA Remover (Toyobo). qPCR was performed using Prism 7900HT or Step One Plus Real-Time PCR Systems (Thermo Fisher) with AmpliTaq Gold® 360 Master Mix (Thermo Fisher), EvaGreen (Biotium), and the primers listed in Table S1; 18S rRNA served as an internal control. Prior to the microarray analysis, a Low Input Quick Amp Labeling Kit (Agilent Technologies) was used to label total RNA that had been purified using the RNeasy MinElute Cleanup Kit (Qiagen). The labeled RNA was then used to probe a SurePrint G3 Mouse Gene Expression 8 × 60K Microarray (Agilent Technologies), and the signals were scanned using a G2565 microarray scanner (Agilent Technologies). Microarray data were extracted from the scanned images using Feature Extraction 10.7 software (Agilent Technologies), and the raw unfiltered microarray data were deposited in the Gene Expression Omnibus dataset (subseries entries GSE149423). The differentially expressed genes were isolated and analyzed using the Subio Platform (Subio).

#### Chromatin immunoprecipitation (ChIP)

MEFs were cross-linked with 1% formaldehyde for 10 min, followed by quenching with 125 mM glycine. Cells were suspended in lysis buffer 1 (50 mM Hepes, 140 mM NaCl, 1 mM EDTA, 10% glycerol, 0.5% NP-40, and 0.25% Triton X-100) supplemented with a protease inhibitor cocktail (Biotool) for 10 min on ice with rotation. The nuclear pellet was washed with lysis buffer 2 (10 mM Tris-HCl, pH 8.0, 200 mM NaCl, 1 mM EDTA, and 0.5 mM EGTA) supplemented with a protease inhibitor cocktail (Biotool). The nuclear pellet was resuspended in lysis buffer 3 (10 mM Tris-HCl, pH 8.0, 300 mM NaCl, 1 mM EDTA, 0.5 mM EGTA, 0.1% sodium deoxycholate, and 0.5% N-laurylsarcosine sodium salt) supplemented with a protease inhibitor cocktail (Biotool) and then sonicated with a Bioruptor for 15 min (30-s pulse and 30-s rest). Debris were removed from the sonicated chromatin by centrifugation, and Triton X-100 was added to a final concentration of 1%. Samples were preincubated with 50% Protein A Sepharose for 2 h. After centrifugation, supernatants were incubated with 3 μL of anti-ATF4 antibodies (Cell signaling technology), 3 μL of anti-DDIT3 antibodies (Proteintech), or 5 μg of control rabbit IgG overnight at 4°C, followed by an incubation with 50% Protein A Sepharose for 2 h. The immunoprecipitates were sequentially washed once each with a low-salt RIPA buffer, a high-salt RIPA buffer, and a LiCl buffer and twice with a Tris-EDTA buffer. The DNA-protein complexes were eluted by heating the samples at 65°C for 6 h. RNA was removed by adding RNase A, and proteins were digested with proteinase K. DNA was collected using the phenol-chloroform method. DNA was quantified using a Step One Plus Real-Time PCR system (Thermo Fisher Scientific) with AmpliTaq Gold® 360 Master Mix (Thermo Fisher), EvaGreen (Biotium), and the primers listed in Table S1.

#### Bioinformatics analysis

Putative ATF4 and DDIT3 binding sites were deduced using Genomatix Gene2Promoter and MatInspector in Genomatix Suite software (Genomatix). Published ChIP-sequencing data (from Series GSE35681, GSE36104 and GSE69309) were downloaded from ChIP-Atlas (Oki et al., 2018) (http://chip-atlas.org) and viewed using the Integrative Genomics Viewer (Broad Institute). Published microarray and mRNA sequencing data were analyzed using GENEVESTIGATOR (Nebion).

### Quantification and statistical analysis

All results are reported as means ± standard deviations (SD). Unpaired two-tailed Student’s t-tests were performed to determine P values for paired samples. One-way ANOVA followed by Holm-Sidak multiple comparisons tests were performed to determine P values for more than three groups. Two-way ANOVA with repeated measurements was performed to analyze kinetic data. All statistical analyses were performed using Excel (Microsoft) or R (R Project) software. *P* values < 0.05 were considered statistically significant.

## Data Availability

• This paper does not report original code. • DNA microarray data generated in this study are deposited with the NCBI Gene Expression Omnibus archive as series GSE149423 and are publicly available as of the date of publication. • Any additional information required to reanalyze the data reported in this paper is available from the lead contact upon request.

## Supplemental information

Document S1. Figures S1–S9 and Table S1

Table S2. Results of microarray analysis, related to Figure 4
