# Supplementary material for: Integrated stress response regulates GDF15 secretion from adipocytes, preferentially suppresses appetite for a high-fat diet and improves obesity
Source: iScience. 2021 Nov 15;24(12):103448. doi: 10.1016/j.isci.2021.103448 (PMC8633987; doi:10.1016/j.isci.2021.103448)
Supplement: Document S1. Figures S1–S9 and Table S1 [file mmc1.pdf]

iScience, Volume *24*

Supplemental information

Integrated stress response regulates GDF15 secretion from adipocytes, preferentially suppresses appetite for a high-fat diet and improves obesity

Masato Miyake, Jun Zhang, Akihiro Yasue, Satoshi Hisanaga, Kazue Tsugawa, Hiroshi Sakaue, Miho Oyadomari, Hiroshi Kiyonari, and Seiichi Oyadomari

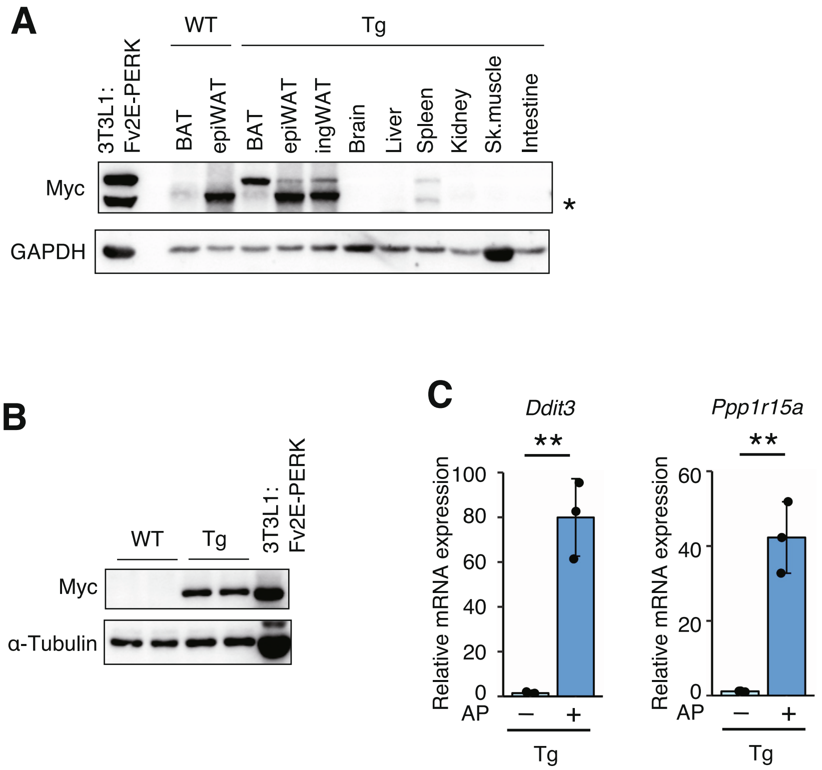

Figure S1 Expression of Fv2E-PERK in Tg mice, related to Figure 1.
(A) Representative immunoblots of Myc (Fv2E-PERK) in BAT and epiWAT of WT mice and in BAT, epiWAT, ingWAT and brain, liver, spleen, kidney, skeletal muscle (gastrocnemius), and small intestine tissues of Tg mice. 3T3L1 cells expressing Fv2E-PERK served as positive controls. N.S., nonspecific band. (B) Representative immunoblots of Myc (Fv2E-PERK) in thioglycolate-treated peritoneal macrophages from WT and Tg mice. 3T3L1 cells expressing Fv2E-PERK served as positive controls. (C) RT-qPCR analysis of Ddit3 and Ppp1r15a mRNA expression in thioglycolate-treated peritoneal macrophages from WT and Tg mice after 12 h of 1 nM AP treatment (vehicle: n = 3, AP: n = 3). All data are presented as the means ± SD. An unpaired two-tailed Student' s t-test was used to analyze the data presented in C.

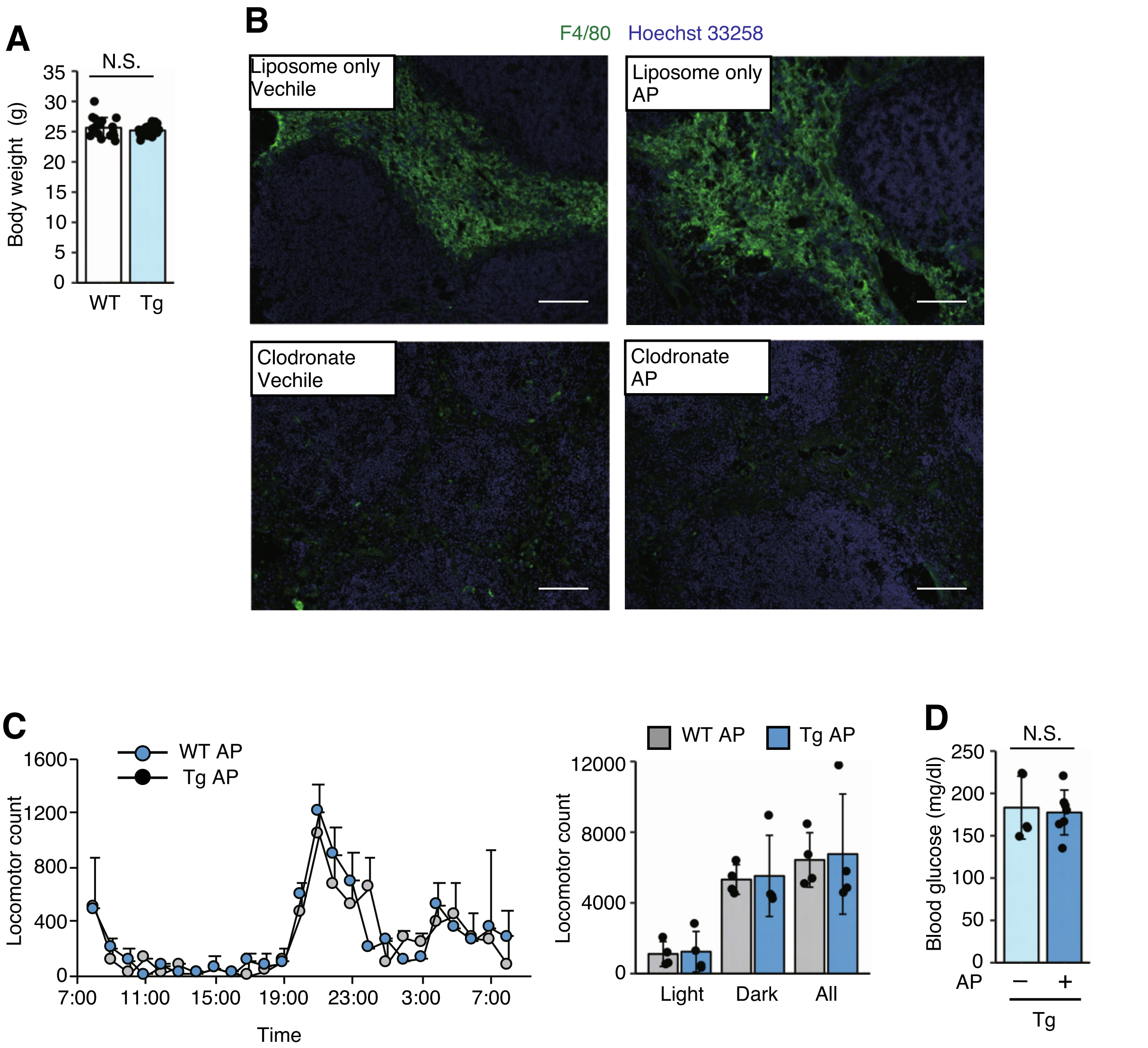

Figure S2. Effects of acute ISR activation in adipocytes, related to Figure 1.
(A) BW of untreated WT and Tg mice at 8 weeks of age (WT: n = 17, Tg: n = 24). (B) Representative photograph of spleen tissue immunostained for the macrophage marker F4/80 from Tg mice treated with control liposomes, clodronate liposomes, vehicle or AP (Bar = 100 μm). (C) Locomotor activity measured immediately and up to 24 h after the injection of AP in WT and Tg mice fed the HFD (WT AP: n = 4, Tg AP: n = 4). (D) Blood glucose concentrations measured 24 h after the vehicle or AP injection in WT and Tg mice fed the HFD (vehicle: n = 5, AP: n = 7). All data are presented as the means ± SD. All data are presented as the mean ± SD. An unpaired two-tailed Student' s t-test was used to analyze the data presented in A, C and D.

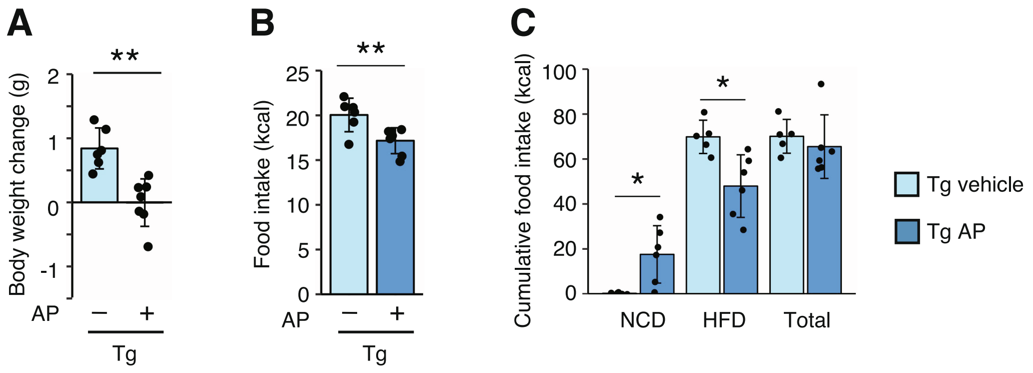

Figure S3. Effects of acute ISR activation in adipocytes on food intake, related to Figure 2.
(A) BW changes 24 h after vehicle or AP injection in female Tg mice fed an HFD (Tg vehicle: n = 5, Tg AP: n = 7). (B) Food intake over 24 h by vehicle- or AP-injected female Tg mice fed a HFD (Tg vehicle: n = 5, Tg vehicle: n = 7). (C) Cumulative intake over 5 days of two diet choices (the NCD and HFD: the suppliers of HFD were different from those of the diets used in other experiments) by vehicle- or AP-injected WT and Tg mice every 24 h (Tg vehicle: n = 5, Tg AP: n = 6). All data are presented as the means ± SD. Unpaired two-tailed Student’ s t-tests were used to analyze the data presented in A, B and C. *P<0.05; **P<0.01.

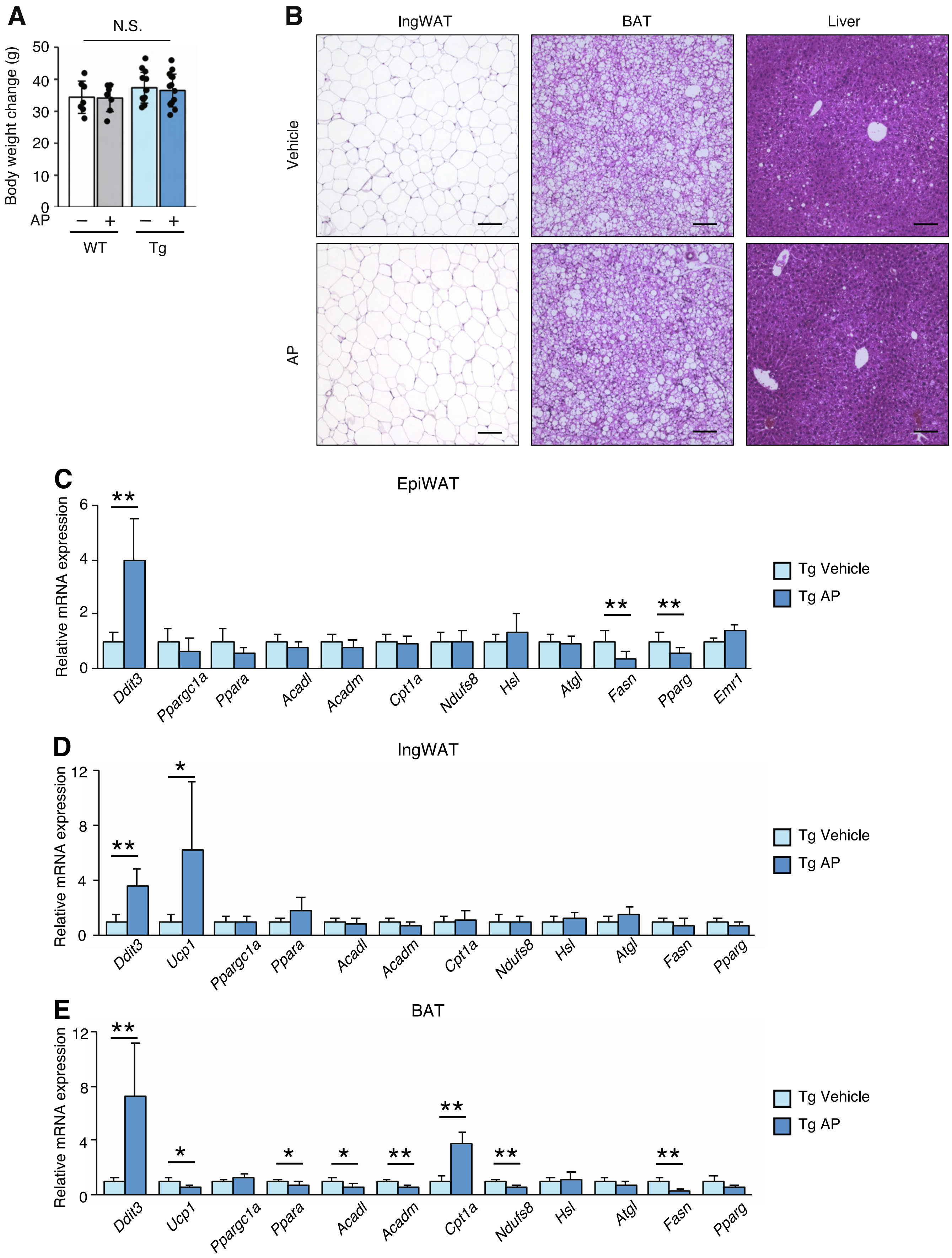
A
N.S.
Body weight change (g)
50
40
30
20
10
0
AP
−
+
−
+
WT
Tg
B
IngWAT
BAT
Liver
Vehicle
AP
C
EpiWAT
Relative mRNA expression
**
**
**
Ddit3
Ppargc1a
Ppara
Acadl
Acadm
Cpt1a
Ndufs8
Hsl
Atgl
Fasn
Pparg
Emr1
Tg Vehicle
Tg AP
D
IngWAT
Relative mRNA expression
**
*
Ddit3
Ucp1
Ppargc1a
Ppara
Acadl
Acadm
Cpt1a
Ndufs8
Hsl
Atgl
Fasn
Pparg
Tg Vehicle
Tg AP
E
BAT
Relative mRNA expression
**
*
*
*
**
**
**
**
Ddit3
Ucp1
Ppargc1a
Ppara
Acadl
Acadm
Cpt1a
Ndufs8
Hsl
Atgl
Fasn
Pparg
Tg Vehicle
Tg AP

Figure S4. Effects of chronic ISR activation in adipocytes, related to Figure 3.

WT and Tg mice were fed a standard diet or an HFD for 8 weeks beginning when they were 4 weeks old and were treated intraperitoneally with vehicle or 0.1 mg/kg AP daily. (A) BW of WT and Tg mice just before the first vehicle or AP treatment after 8 weeks of HFD feeding (WT vehicle: n = 7, WT AP: n = 7, Tg vehicle: n = 13, Tg AP: n = 15). (B) Representative photograph of HE staining of ingWAT, BAT and liver tissue from vehicle- or AP-injected Tg mice fed an HFD (Bar = 100 μm). (C-E) RT-qPCR analysis of metabolism-related mRNA expression in the indicated tissues of vehicle- or AP-injected Tg mice fed an HFD (Tg vehicle: n = 6, Tg AP: n = 6). All data are presented as the mean ± SD. Unpaired two-tailed Student' s t-tests were used to analyze the data presented in C, D and E. One-way ANOVA followed by Holm-Sidak multiple comparisons tests were used to analyze the data presented in A. *P<0.05; **P<0.01.

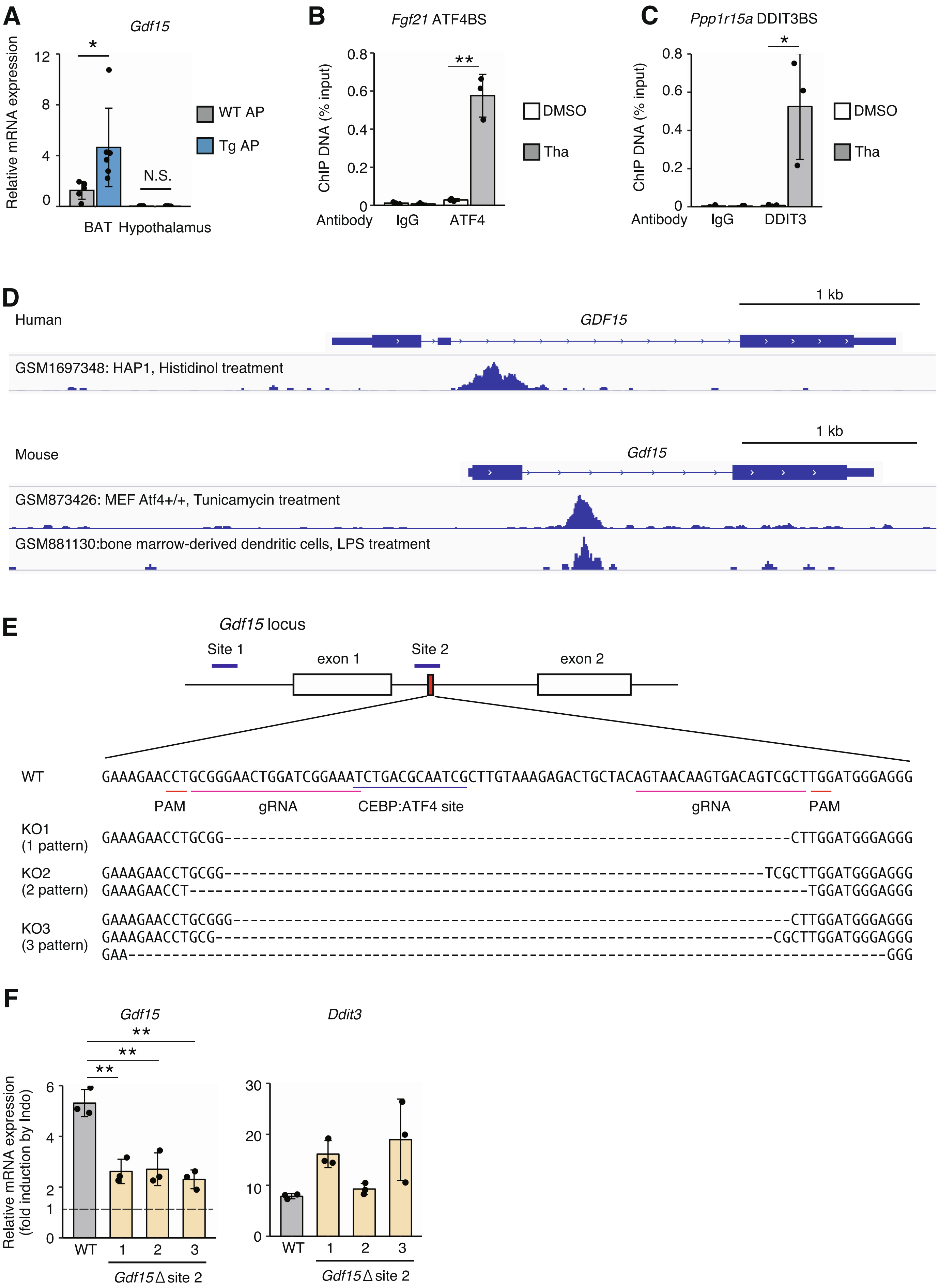

A
Gdf15
Relative mRNA expression
12
8
4
0
*
N.S.
BAT
Hypothalamus
WT AP
Tg AP
B
Fgf21 ATF4BS
ChIP DNA (% input)
0.8
0.6
0.4
0.2
0
**
Antibody
IgG
ATF4
DMSO
Tha
C
Ppp1r15a DDIT3BS
ChIP DNA (% input)
0.8
0.6
0.4
0.2
0
*
Antibody
IgG
DDIT3
DMSO
Tha
D
Human
1 kb
GDF15
GSM1697348: HAP1, Histidinol treatment
Mouse
1 kb
Gdf15
GSM873426: MEF Atf4+/+, Tunicamycin treatment
GSM881130:bone marrow-derived dendritic cells, LPS treatment
E
Gdf15 locus
Site 1
exon 1
Site 2
exon 2
WT GAAAGAACCTGCGGGAACTGGATCGGAAATCTGACGCAATCGCTTGTAAAGAGACTGCTACAGTAACAAGTGACAGTCGCTTGGATGGGAGGG
PAM
gRNA
CEBP:ATF4 site
gRNA
PAM
KO1 (1 pattern) GAAAGAACCTGCGG--CTTGGATGGGAGGG
KO2 (2 pattern) GAAAGAACCTGCGG---TCGCTTGGATGGGAGGG
GAAAGAACCT--TGGATGGGAGGG
KO3 (3 pattern) GAAAGAACCTGCGGG---CTTGGATGGGAGGG
GAAAGAACCTGCG--CGCTTGGATGGGAGGG
GAA---GGG
F
Gdf15
**
**
**
Relative mRNA expression (fold induction by Indo)
6
4
2
1
0
WT
1
2
3
Gdf15Δ site 2
Ddit3
30
20
10
0
WT
1
2
3
Gdf15Δ site 2

Figure S5. Regulation of GDF15 expression by ATF4 and DDIT3, related to Figure 4.
(A) RT-qPCR analysis of Gdf15 mRNA expression in the BAT and hypothlumus of AP-treated WT or Tg mice 24 h after the injection (WT AP: n = 5, Tg AP: n = 6). (B) ChIP-qPCR analysis of ATF4 occupancy at the Fgf21 promoter in MEFs 8 h after thapsigargin (Tha) treatment (n = 3). (C) ChIP-qPCR analysis of DDIT3 occupancy at the Ppp1r15a promoter in MEFs 8 h after Tha treatment (n = 3). (D) ATF4 occupancy at the Gdf15 locus derived from previously published ChIP-seq data on humans and mice treated with an ISR-inducing stressor (Histidinol, Tunicamycin and LPS). (E) Strategy for depletion of CEBP:ATF4 binding sites in the Gdf15 locus using CRISPR/Cas9 and the actual sequence of the established clone. (F) RT-qPCR analysis of Gdf15 and Ddit3 mRNA expression in MEFs with deleted CEBP:ATF4 binding sites at 10 h after treatment with indometacin (indo: n = 3). All data are presented as the mean ± SD. Unpaired two-tailed Student' s t-tests were used to analyze the data presented in A, B and C. One-way ANOVA followed by Holm-Sidak multiple comparisons tests were used to analyze the data presented in F. *P<0.05; **P<0.01.

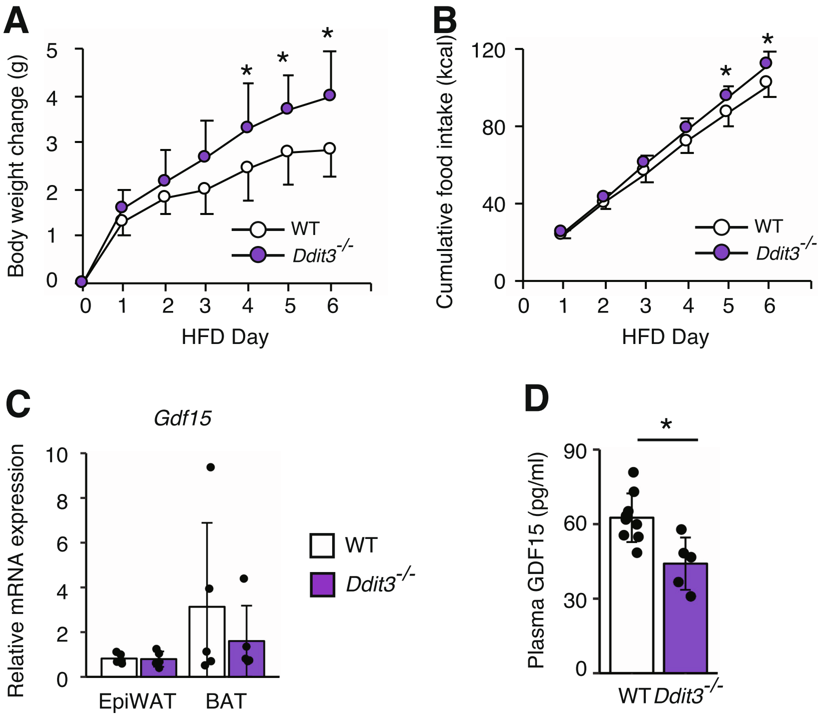

Figure S6. Phenotype of Ddit3 knockout mice, related to Figure 4.
(A) BW changes of WT and Ddit3-/- mice after switching to the HFD (WT: n = 7, Ddit3-/-: n = 7). (B) Cumulative food intake by WT and Ddit3-/- mice after switching to the HFD (WT: n = 7, Ddit3-/-: n = 7). (C) RT-qPCR analysis of Gdf15 mRNA expression in epiWAT and BAT of WT and Ddit3-/- mice after 7 days of HFD feeding (WT: n = 5, Ddit3-/-: n = 5). (D) Plasma GDF15 concentrations in WT and Ddit3-/- mice after 7 days of HFD feeding (WT: n = 9, Ddit3-/-: n = 5). All data are presented as the means ± SD. Unpaired two-tailed Student' s t-tests were used to analyze the data presented in A, B, C and D. *P<0.05; **P<0.01.

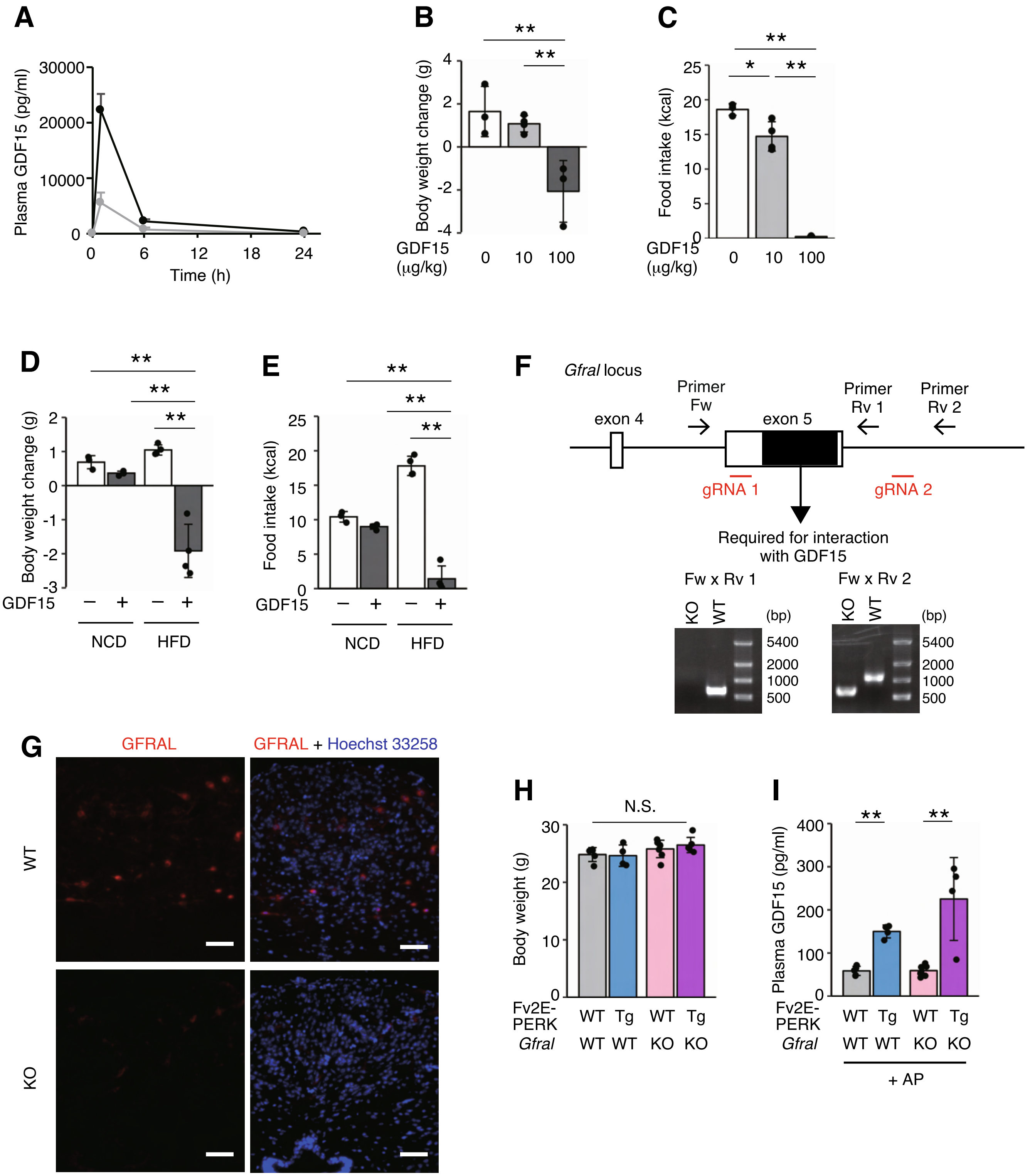
A
Plasma GDF15 (pg/ml)
Time (h)
B
Body weight change (g)
GDF15 (µg/kg)
0 10 100
C
Food intake (kcal)
GDF15 (µg/kg)
0 10 100
D
Body weight change (g)
GDF15
NCD HFD
E
Food intake (kcal)
GDF15
NCD HFD
F
Gfral locus
exon 4
Primer Fw
exon 5
Primer Rv 1
Primer Rv 2
gRNA 1
gRNA 2
Required for interaction with GDF15
Fw x Rv 1
Fw x Rv 2
KO WT
(bp)
5400
2000
1000
500
G
GFRAL
GFRAL + Hoechst 33258
WT
KO
H
N.S.
Body weight (g)
Fv2E-PERK
Gfral
WT Tg WT Tg
WT WT KO KO
I
Plasma GDF15 (pg/ml)
Fv2E-PERK
Gfral
WT Tg WT Tg
WT WT KO KO
+ AP

Figure S7. Role of the GDF15-GFRAL pathway in the adipocyte ISR activation, related to Figure 5. (A) Plasma GDF15 concentrations after rhGDF15 injected WT at indicated time (n = 4). (B) BW changes of WT mice treated with the indicated dose of GDF15 after switching to the HFD after 12 h (saline: n = 3, 0.01 mg/kg: n = 4, 0.1 mg/kg: n = 4). (C) Food intake by WT mice treated with the indicated dose of GDF15 after switching to the HFD after 12 h (saline: n = 3, 0.01 mg/kg: n = 4, 0.1 mg/kg: n = 4). (D) BW changes of WT mice treated with 0.03 mg/kg of GDF15 fed the NCD or the HFD after 12 h (saline NCD: n = 3, GDF15 NCD: n = 4, saline HFD: n = 4, GDF15 HFD: n = 4). (E) Food intake by saline- or GDF15-injected WT mice fed the NCD or the HFD was measured for 12 h (saline NCD: n = 3, GDF15 NCD: n = 4, saline HFD: n = 4, GDF15 HFD: n = 4). (F) Strategy for generation of Gfral knockout (KO) mice using CRISPR/Cas9 and the PCR results obtained for genomic DNA of WT and Gfral KO mice using the indicated primer pairs. (G) Representative photograph of area postrema immunostained for GFRAL from WT and Gfral KO mice (Bar = 50 μm) (WT: n = 3, Gfral KO: n = 2). (H) BWs of WT, Tg, Gfral KO, and Tg:Gfral KO mice at 8 weeks of age (WT: n = 5, Tg: n = 4, Gfral KO: n = 7, Tg:Gfral KO: n = 6). (I) Plasma GDF15 concentrations in WT, Tg, Gfral KO, and Tg:Gfral KO mice 24 h after AP injection (WT: n = 5, Tg: n = 4, Gfral KO: n = 6, Tg:Gfral KO: n = 4). All data are presented as the means ± SD. One-way ANOVA followed by Holm-Sidak multiple comparisons tests were used to analyze the data presented in B, C, D, E, H and I. *P<0.05; **P<0.01.

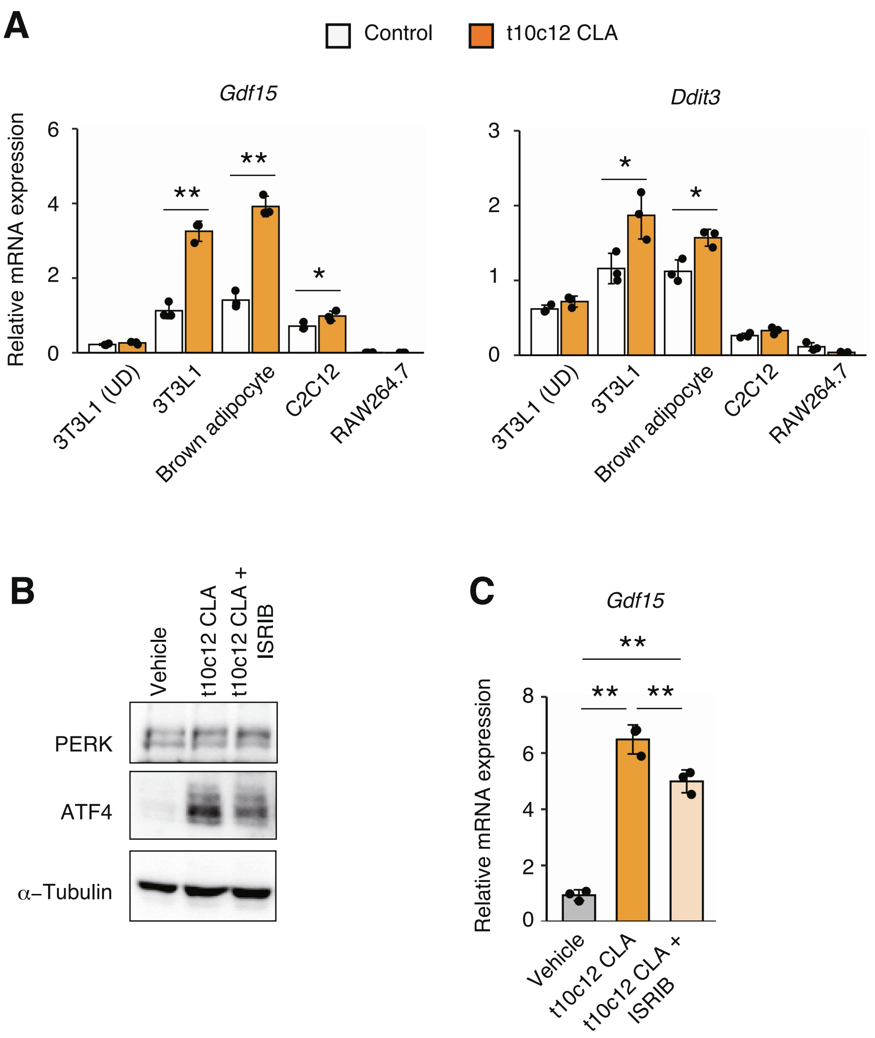

Figure S8. Effects of 10(E),12(Z)-octadecadienoic acid on the ISR and GDF15 expression, related to Figure 6.

(A) RT-qPCR analysis of Gdf15 and Ddit3 mRNA expression in undifferentiated 3T3L1 (3T3L1 UD) cells, differentiated 3T3L1 cells, differentiated brown adipocytes, differentiated C2C12 cells, and RAW264.7 cells (n = 3). (B) Representative immunoblots of PERK, ATF4 and α-tubulin in 3T3L1 cells treated with 10(E),12(Z)-octadecadienoic acid and ISRIB (n = 3). (C) RT-qPCR analysis of Gdf15 mRNA expression in 3T3L1 cells treated with 10(E),12(Z)-octadecadienoic acid and ISRIB (n = 3). All data are presented as the means ± SD. Unpaired two-tailed Student' s t-tests were used to analyze the data presented in A. One-way ANOVA followed by Holm-Sidak multiple comparisons tests were used to analyze the data presented in C. *P<0.05; **P<0.01.

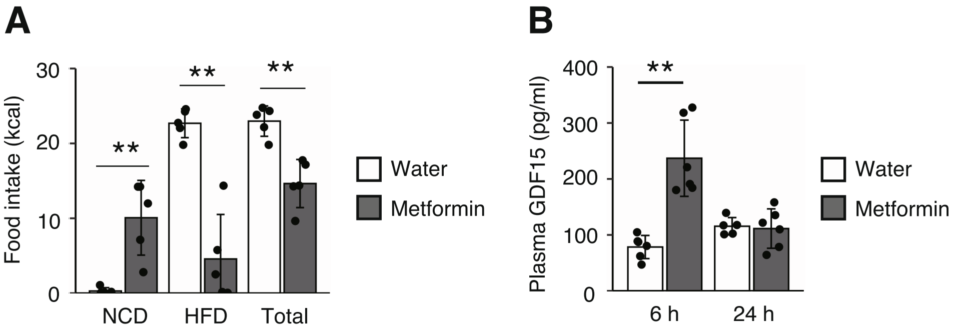

Figure S9. Effect of metformin on food choice between the NCD and the HFD, related to Discussion

(A) Food intake measured for 24 h during the two-diet choice (NCD and HFD) experiment using water- or metformin-injected WT mice (water: n = 5, metformin: n = 5). (B) Plasma GDF15 concentrations after water or metformin injected WT at indicated time (water at 8 h: n = 6, metformin at 8 h: n = 6 water at 24 h: n = 5, metformin at 24 h: n = 6). Data are presented as the means ± SD. Unpaired two-tailed Student’ s t-tests were used to analyze the data.

Table S1 DNA and RNA sequence used in this study, related to STAR Methods

Primer list in RT-qPCR

Gene name	Sense sequence (5' - 3')	Antisense sequence (5' - 3')
Gdf15	GAGCTACGGGGTCGCTTC	GGGACCCCAATCTCACCT
Chop	GCGACAGAGCCAGAATAACA	GATGCACTTCCTTCTGGAACA
Trib3	CGCTTTGTCTTCAGCAACTGT	TCATCTGATCCAGTCATCACG
Hspa5	TCATGACATTCAGTCCAGCAA	CTGAGGCGTATTTGGGAAAG
Dnajb9	CACAAAGATGCCTTTTCTACCG	TTAAACTTTTCAGCTTAATGACGTG
Edem1	GGTCTTCGAAGCTACGATAAGG	GGGCTGTTTGGAATCAGTTATTA
Atf3	GACAGAGTGCCTGCAGAAAGA	CATGTATATCAAATGCTGTTTCTCATT
Ppp1r15a	ACGATCGCTTTTGGCAAC	GACATGCTGGGGTCTTGG
Ppargc1a	CCGTAAATCTGCGGGATGATG	CAGTTTCGTTCGACCTGCGTAA
Ppara	TCGGCAAAGACAAATGCTTCA	CAGTGCCTTGTGCCAACCA
Acadl	TGCCCTCCGCCCGATGTTCTCATTC	TGCGATGTTGATGCCAAGCAAGCC
Acadm	TGATGTGGCGGCCATTAAGA	GGGTTAGAACGTGCCAACAAGAA
Cpt1a	CCCAAACCCACCAGGCTACA	AGAGCCGAGTCATGGAAGCC
Ndufs8	CCTCTTTCGAGAGCCTGCCA	GTCATAGCGTGTCGTTCGGC
Hsl	AGCGCTGGAGGAGTGTTTT	CCGCTCTCCAGTTGAACC
Atgl	TGACCATCTGCCTTCCAGA	TGTAGGTGGCGCAAGACA
Fasn	AGCACTGCCTTCGGTTCAGTC	AAGAGCTGTGGAGGCCACTTG
Pparg	CTCCTGTTGACCCAGAGCAT	GGATCCGGCAGTTAAGATCA
Emr1	TTTCCTCGCCTGCTTCTTC	CCCCGTCTCTGTATTCAACC
Ucp1	ACTGCCAAAGTCCGCCTTCAGATCC	AGGCTGCCCAATGAACACTGCCA
18S	GTAACCCGTTGAACCCCATT	CCATCCAATCGGTAGTAGCG

Primer list in ChIP-qPCR

Gene name	Sense sequence (5' - 3')	Antisense sequence (5' - 3')
Gdf15 site1	GCTGTGCCTCCTCACAGAGT	CCCGCCATCTCCTTTGTCCA
Gdf15 site2	ACGGAAAGAACCTGCGGGAA	CCTCCCATCCAAGCGACTGT
Fgf21	GACTGCAGGAAACAACCCAGC	TTAGCATTCGGGCCTTGTGC
Ppp1r15a	GCCTGATCAGCCACGGATACTC	CGCGAACCTTATCTGGCAGTC

Oligo DNA list in CRISPR/Cas9 genome editing for cell culture

Gene name	Sense sequence (5' - 3')	Antisense sequence (5' - 3')
Ddit3	CACCGCTTGGAGACGGTGTCCAGCT	AAACAGCTGGACACCGTCTCCAAGC
Atf4	CACCCCCAAACCCGACTGGTCGAA	AAACTTCGACCAGTCGGGTTTGGG
Gdf15 1	CACCGATTTCCGATCCAGTTCCCGC	AAACGCGGGAACTGGATCGGAAATC
Gdf15 2	CACCGAGTAACAAGTGACAGTCGCT	AAACAGCGACTGTCACTTGTTACTC

crRNA list in CRISPR/Cas9 genome editing for mouse

Gene name	Sequence (5' - 3')
Gfral 1	GGTGACAGAGGCGTGTGTAG
Gfral 2	AACTTTGAAAGATGATATAC

Primer list in mouse genotyping

Gene name	Sequence (5' - 3')
Tg Fw	AGTCATTAGGGGATGGGAGG
Tg Rv	CCGTCTCCTGGGGAGATAGT
Gdf15 Fw1	CTCCACTTTGGTCCTGTCCATTC
Gdf15 Rv1	ATGACGGTGGGAACTCTAGCAA
Gdf15 Rv2	AGACTTTTCCACACAACAGGC
